# Gut microbial dysbiosis correlates with stroke severity markers in aged rats

**DOI:** 10.3389/fstro.2022.1026066

**Published:** 2022-12-21

**Authors:** Tyler C. Hammond, Sarah Messmer, Jacqueline A. Frank, Doug Lukins, Rita Colwell, Ai-Ling Lin, Keith R. Pennypacker

**Affiliations:** ^1^Lin Brain Lab, Department of Neuroscience, Sanders-Brown Center on Aging, University of Kentucky, Lexington, KY, United States; ^2^Department of Neurology, The Center for Advanced Translational Stroke Science, University of Kentucky, Lexington, KY, United States; ^3^Department of Radiology, University of Kentucky, Lexington, KY, United States; ^4^CosmosID Inc, Rockville, MD, United States; ^5^Division of Biological Sciences and Institute for Data Science and Informatics, Department of Radiology, University of Missouri, Columbia, MO, United States

**Keywords:** stroke, microbiome, inflammation, imaging, aging

## Abstract

**Background:**

An imbalanced gut microbial community, or dysbiosis, has been shown to occur following stroke. It is possible that this dysbiosis negatively impacts stroke recovery and rehabilitation. Species level resolution measurements of the gut microbiome following stroke are needed to develop and test precision interventions such as probiotic or fecal microbiota transplant therapies that target the gut microbiome. Previous studies have used 16S rRNA amplicon sequencing in young male mice to obtain broad profiling of the gut microbiome at the genus level following stroke, but further investigations will be needed with whole genome shotgun sequencing in aged rats of both sexes to obtain species level resolution in a model which will better translate to the demographics of human stroke patients.

**Methods:**

Thirty-nine aged male and female rats underwent middle cerebral artery occlusion. Fecal samples were collected before stroke and 3 days post stroke to measure gut microbiome. Machine learning was used to identify the top ranked bacteria which were changed following stroke. MRI imaging was used to obtain infarct and edema size and cerebral blood flow (CBF). ELISA was used to obtain inflammatory markers.

**Results:**

Dysbiosis was demonstrated by an increase in pathogenic bacteria such as *Butyricimonas virosa* (15.52 fold change, *p* < 0.0001), *Bacteroides vulgatus* (7.36 fold change, *p* < 0.0001), and *Escherichia coli* (47.67 fold change, *p* < 0.0001). These bacteria were positively associated with infarct and edema size and with the inflammatory markers Ccl19, Ccl24, IL17a, IL3, and complement C5; they were negatively correlated with CBF. Conversely, beneficial bacteria such as *Ruminococcus flavefaciens* (0.14 fold change, *p* < 0.0001), *Akkermansia muciniphila* (0.78 fold change, *p* < 0.0001), and *Lactobacillus murinus* (0.40 fold change, *p* < 0.0001) were decreased following stroke and associated with all the previous parameters in the opposite direction of the pathogenic species. There were not significant microbiome differences between the sexes.

**Conclusion:**

The species level resolution measurements found here can be used as a foundation to develop and test precision interventions targeting the gut microbiome following stroke. Probiotics that include *Ruminococcus flavefaciens, Akkermansia muciniphila, and Lactobacillus murinus* should be developed to target the deficit following stroke to measure the impact on stroke severity.

## 1. Introduction

Over 795,000 people suffer a stroke every year in the United States alone (Benjamin et al., 2017). Recent advances in acute stroke therapies have lowered stroke mortality, but survivors are often left with severely impaired motor and cognitive performance (Moy et al., 2017). Rehabilitation therapies are beneficial at inducing neuroplasticity to overcome these impairments, but over 40% of stroke survivors are left with moderate to severe disabilities that markedly reduce quality of life (Carandang et al., 2006). Novel multimodal approaches are needed to promote plasticity and sensorimotor function through a combination of current rehabilitation therapies with other treatments designed to foster neuroplasticity.

Accumulating evidence suggests that gut microbes modulate brain plasticity *via* the bidirectional gut-brain axis and may play a role in stroke rehabilitation (Leung and Thuret, 2015). A severely imbalanced microbial community, or dysbiosis, has been shown to occur following stroke, causing a systemic flood of neuro- and immunomodulatory substances due to increased gut permeability and decreased gut motility (Stanley et al., 2018). These substances can impact neuroinflammation as commensal bacteria invade the bloodstream and as intestinal lymphocytes migrate from gut-associated lymphoid tissue to the brain (Singh et al., 2016). Fecal microbiota transplant has been shown to normalize brain lesion-induced dysbiosis and to improve stroke outcome in mice (Singh et al., 2016). The microbiome is modifiable as it is influenced by environmental factors such as diet and exercise and could potentially be a therapeutic target in stroke rehabilitation through special nutritional and pharmacological interventions and physical therapies (Winek et al., 2016; Mailing et al., 2019). To our knowledge, no studies have measured the species level resolution necessary to develop precision interventions such as probiotics or fecal microbiota transplants that target the gut microbiota following stroke. Furthermore, no microbiome studies have been performed on aged rats of both sexes, which are better matched to the demographics of the average human stroke patient and better reflect the microbiome changes we know to occur with age than the young male mice used in most studies (Spychala et al., 2018). The microbiome changes found in this study need to be examined and correlated with clinical imaging markers of stroke and inflammatory markers to understand better whether the microbiome could be a therapeutic target in stroke rehabilitation.

Here we identify the gut-brain axis changes that occur following stroke in aged rats using high resolution whole genome shotgun sequencing and correlate them with clinical imaging markers of stroke including MRI-based infarct size, edema size, and cerebral blood flow (CBF) as well as inflammatory markers. We found that microbial communities are disrupted in an aged rat population following stroke, showing significantly different beta diversity, increased alpha diversity, and changes in the relative abundance of 5 of the 6 major phyla found in the gut. Changes in 13 bacterial species as detected by machine learning were highly associated with stroke and changes in these species were also associated with increased infarct and edema size and decreased CBF. Changes in the microbiome due to stroke were also associated with increases in 49 inflammatory markers. Additionally our previous studies found that leukemia inhibitory factor (LIF) inhibits the peripheral immune response to stroke (Davis et al., 2020). Therefore, we sought to determine the effect of LIF on the microbiome following stroke.

## 2. Results

**Figure 1:**
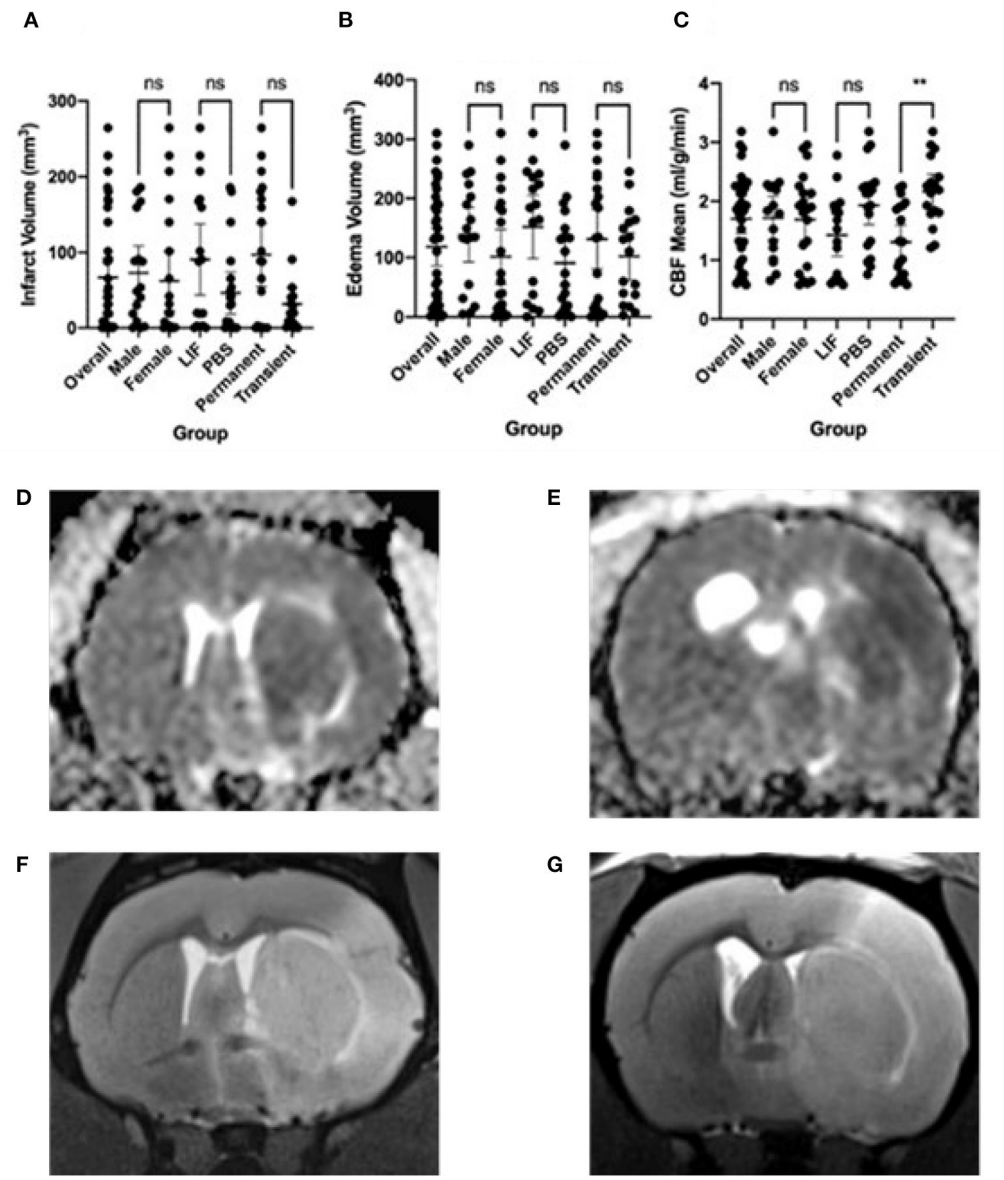
Imaging features following stroke. **(A)** Infarct volume, **(B)** Edema volume, and **(C)** Cerebral Blood Flow (CBF) mean following stroke in overall rats and separated by male, female, LIF, PBS, Permanent, and Transient. Representative images are: **(D)** Edema from Permanent MCAO, **(E)** Edema from Transient MCAO, **(F)** Infarct from Permanent MCAO, **(G)** Infarct from Transient MCAO Groups were compared using a Mann-Whitney test. * <0.05 ** <0.01.

**Figure 2:**
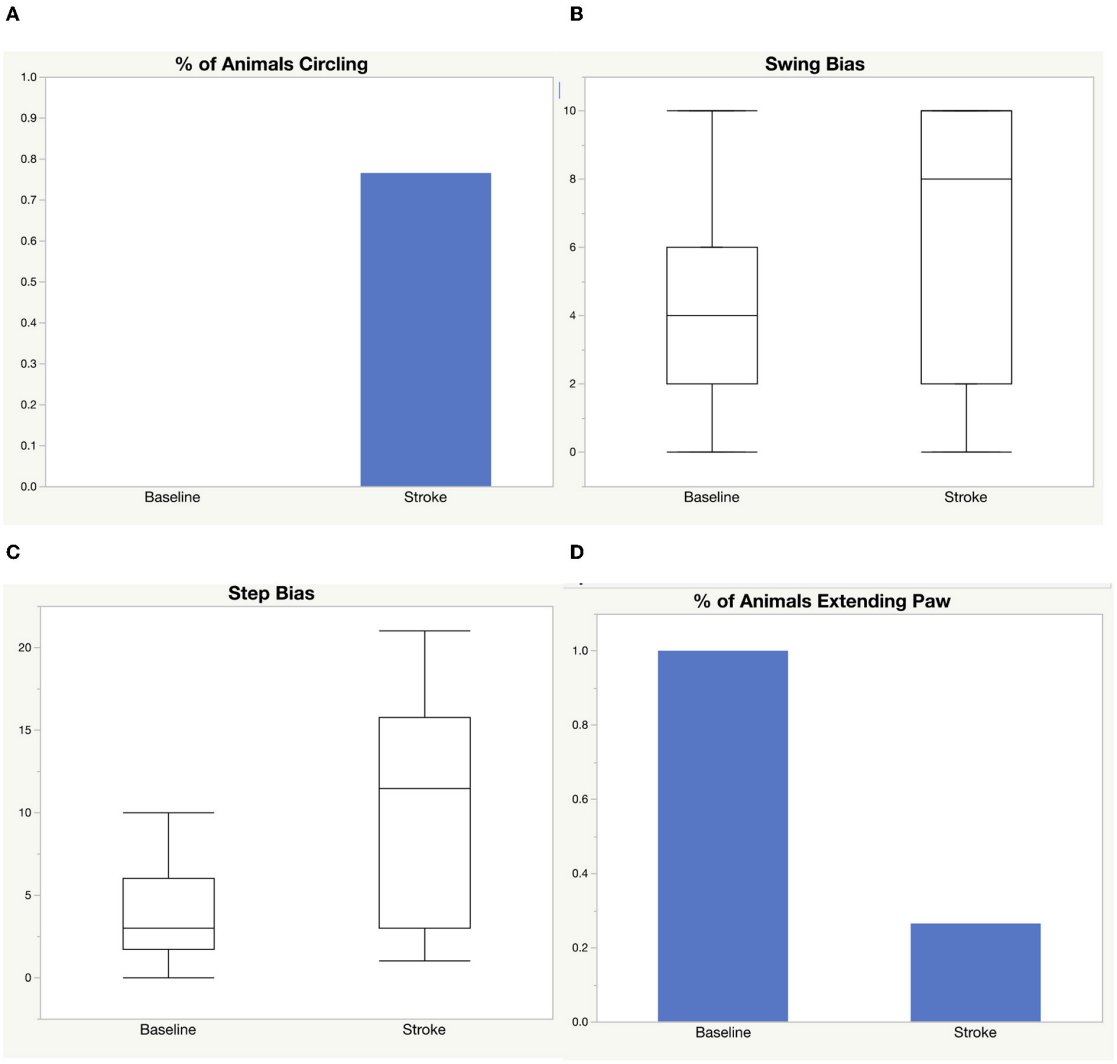
Motor function skills before and after stroke. Four tests were used to measure motor function before and after stroke: **(A)** circling, **(B)** elevated body swing test, **(C)** step test, **(D)** paw extension test.

We performed a middle cerebral artery occlusion on a sample of 39 aged rats of both sexes (age 15–18 months). Half of the rats underwent a permanent occlusion and half underwent a transient 5-h occlusion. We analyzed male and female rats. We analyzed all rats before and after middle cerebral artery occlusion and considered sex, surgery type, and treatment with LIF or PBS in the analysis. We administered a LIF treatment on half of the rats based on previous work suggesting that LIF is an anti-inflammatory that regulates the immune/inflammatory response to stroke (Davis et al., 2020). The rats had an average of 96.50 mm^3^ infarct size, 131.0 mm^3^ edema size, and 1.31 ml/g/min CBF from a permanent occlusion and 31.46 mm^3^ infarct size, 102.1 mm^3^ edema size, and 2.16 ml/g/min CBF from a transient occlusion. Infarct and edema volumes were not significantly different between sex, treatment group, or occlusion type. No significant difference in CBF was detected between sex or treatment, but, as expected, a significant difference occurred between permanent and transient occlusion in CBF (Figure 1). We used four tests to determine motor function skills before and after stroke. 76% of the rats circled following stroke (Figure 2A), there was a swing bias of 8 (Figure 2B), there was a step bias of 9.3 (Figure 2C), and only 26.5% of the rats extended their paw (Figure 2D).

### 2.1. The aged rat gut microbiome is disrupted following stroke

**Figure 3:**
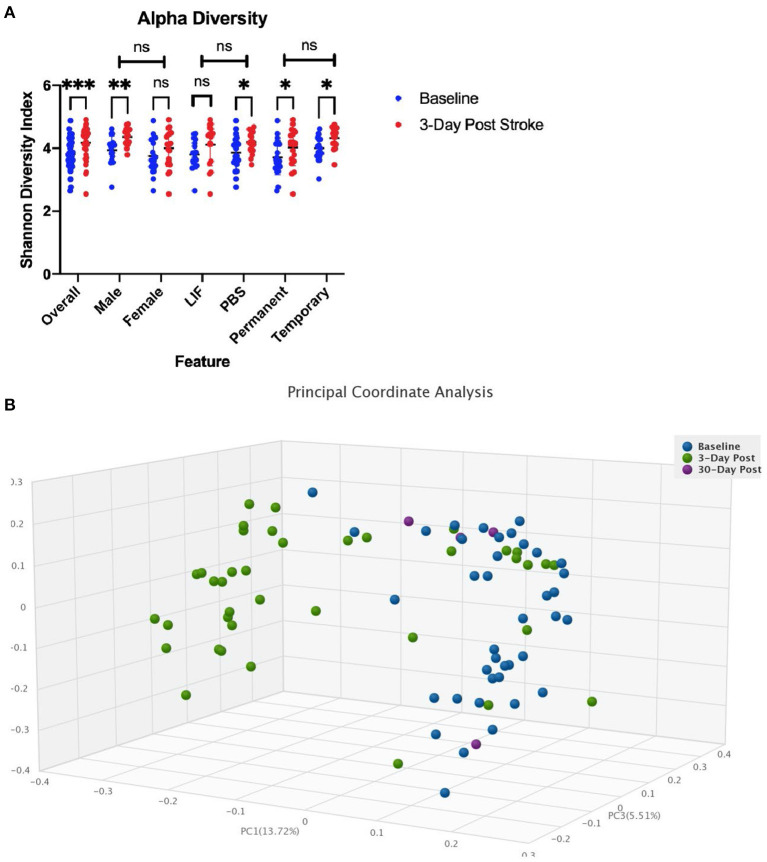
Diversity changes following stroke. **(A)** Alpha diversity as measured by the Shannon diversity index detecting species richness and evenness is increased following stroke. There is no difference in change across sex, treatment, or stroke type. **(B)** Beta Diversity as measured by Bray-Curtis method comparing how dissimilar samples are. * < 0.05, ** < 0.01, *** < 0.0001.

We performed an analysis on the gut microbial communities of the rats by running whole genome shotgun sequencing using DNA quantification services provided by CosmosID. We collected fecal samples 24 h before stroke and within 72 h following stroke. Comparing the alpha diversity before and after stroke, we found that richness and evenness increased from 3.818 on the Shannon diversity index (Longuet-Higgins, 1971) to 4.178 (Figure 3A). There were no differences in the change of alpha diversity between sex, treatment, or occlusion type. Comparing the beta diversity before and after stroke, we found that the microbial communities were significantly different between baseline and stroke (*p* = 0.0001), but no significant microbial community differences were detected based on sex, treatment, or occlusion type (Figure 3B; Supplementary Table 1).

**Figure 4:**
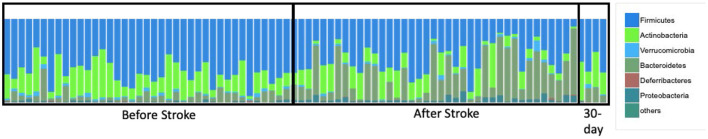
Phyla changes as a result of stroke. Relative abundance shows phyla composition before and after stroke.

We investigated specific differences in the relative abundance of the major bacterial phyla in the gut (Figure 4). We found increases in proteobacteria and Bacteroidetes and decreases in firmicutes, verrucomicrobia, and actinobacteria following stroke (Supplementary Table 2A). This translates to a sharp decrease in the firmicutes to bacteroidetes ratio. Using linear regression, the major bacterial phyla predict infarct size with an *R*^2^= 0.3866 and edema size with an *R*^2^= 0.6022 (Supplementary Table 2B).

### 2.2. The top 13 disrupted bacterial species following stroke

We investigated specific differences in the relative abundance of the major bacterial species in the gut. There was a total of 29 species increased and 23 species decreased following stroke (Table 1). Supplementary Table 3 gives a detailed description of all the taxa that were increased (red) or decreased (green) following stroke. Using random forest machine learning classification, we found the most important bacterial species that predict stroke verse baseline with an 85.14% accuracy. They include an increase in *Butyricimonas virosa, Bacteroides vulgatus, Escherichia coli, Bacteroides uniformis, Bacteroides dorei, Parabacteroides distasonis*, and *Alistipes indistinctus* and a decrease in *Ruminococcus flavefaciens, Akkermansia muciniphila, Ruminococcus_u_s, [Clostridium] clostridioforme, Lactobacillus murinus*, and *Lachnospiraceae bacterium 3-1*. Using linear regression with backwards elimination (Table 2), we found that increases in *Ruminococcus_u_s* and *Alistipes indistinctus* and decreases in *Lachnospiraceae bacterium 3-1* predict infarct volume with an *R*^2^= 0.4433. Increases in *Butyricinomas virosa, Bacteroides uniformis*, and *Ruminococcus_u_s* and decreases in *Ruminococcus flavefaciens* predict edema with an *R*^2^= 0.6230. Finally, decreases in *Alistipes indistinctus* predict CBF with an *R*^2^ = 0.1825.

**Table 1:** Top 13 bacterial species changes following stroke as detected by random forest.

**Bacterial species**	**Fold change in relative abundance**	**Gram stain**	**Phylum**
*Butyricimonas virosa*	15.52	(-)	Bacteroides
*Ruminococcus flavefaciens*	0.14	(+)	Firmicutes
*Akkermansia muciniphila*	0.78	(-)	Verrucomicrobia
*Bacteroides vulgatus*	7.36	(-)	Bacteroidetes
*Escherichia coli*	47.67	(-)	Proteobacteria
*Bacteroides uniformis*	5.15	(-)	Bacteroidetes
*Bacteroides dorei*	5.29	(-)	Bacteroidetes
*Ruminococcus_u_s*	0.25	(+)	Firmicutes
*Parabacteroides distasonis*	10.84	(-)	Bacteroidetes
*[Clostridium] clostridioforme*	0.42	(+)	Firmicutes
*Alistipes indistinctus*	335.73	(-)	Bacteroidetes
*Lactobacillus murinus*	0.36	(+)	Firmicutes
*Lachnospiraceae bacterium 3-1*	0.40	(+)	Firmicutes

Predictive accuracy of random forest model: 85.14%.

**Table 2:** Linear regression models predicting infarct, edema, and cerebral blood flow (CBF) by top 13 species.

**Parameter**	**Estimate**	**DF**	**SS**	***F*-Ratio**	***P*-value**
**Predicting infarct**					
Intercept	10.41	1	0	0	1
*Ruminococcus_u_s*	5.34	1	201.66	11.85	0.0015
*Alistipes indistinctus*	3.05	1	122.20	7.18	0.0112
*Lachnospiraceae bacterium 3-1*	−24.97	1	141.01	8.28	0.0068
**Predicting edema**					
Intercept	3.05	1	0	0	1
*Butyricimonas virosa*	35.48	1	73.77	5.68	0.0229
*Ruminococcus flavefaciens*	−6.50	1	276.79	21.30	<0.0001
*Bacteroides uniformis*	12.93	1	59.99	4.62	0.0394
*Ruminococcus_u_s*	4.92	1	152.28	11.72	0.0016
**Predicting cerebral blood flow**					
Intercept	1.48	1	0	0	1
*Alistipes indistinctus*	−0.46	1	3.53	7.82	0.008

Model parameters selected using backward elimination from top 13 list in Table 1.

We investigated potential interactions between bacterial species in predicting infarct size, edema size, and CBF (Supplementary Table 4). Using a feasible solution algorithm (FSA) for finding interactions, we found that decreases in *Lachnospiraceae bacterium A2* and *Lactobacillus murinus* predict infarct size, but a combination of the two predicts a dramatic increase in the prediction value with an *R*^2^= 0.6206. Decreases in *Lachnospiraceae bacterium A4* and *Lactobacillus murinus* predict edema size, but a combination of the two have stronger predictive ability with an *R*^2^= 0.6454. Decreases in *Adlercreutzia equolifaciens* and *Desulfovibrio desulfuricans* predict CBF, but again, a combination of the two has a stronger prediction with an *R*^2^= 0.8093.

### 2.3. Bacterial community disruptions following stroke are correlated with stroke severity markers

We investigated the correlation of all the bacterial species with infarct size and edema size (Tables 3, 4). Using the MaAsLin 2 R package (Mallick et al., 2021), which automatically normalizes and transforms all variables in preparation for linear regression, we correlated metagenomic sequencing with imaging variables of stroke severity. Twenty-seven bacterial species were positively correlated and 19 negatively correlated with infarct volume. Thirty species were positively correlated, and 31 species were negatively correlated with edema volume. No species were correlated with CBF.

**Table 3:** Correlation of bacterial species with infarct and edema.

**Bacteria**	**Coefficient**	**Bacteria**	**Coefficient**
**Species increase associated with infarct**		**Species increase associated with edema**	
*Alistipes finegoldii*	0.32	*Alistipes finegoldii*	0.35
*Alistipes indistinctus*	0.29	*Alistipes indistinctus*	0.34
*Alistipe sp HGB5*	0.33	*Alistipes sp HGB5*	0.38
*Alistipes timonensis*	0.16	*Alistipes timonensis*	0.17
*Bacteroides caccae*	0.29	*Bacteroides caccae*	0.32
*Bacteroides dorei*	0.38	*Bacteroides dorei*	0.43
*Bacteroides eggerthii*	0.30	*Bacteroides eggerthii*	0.34
*Bacteroides fragilis*	0.09	*Bacteroides fragilis*	0.11
*Bacteroides intestinalis*	0.17	*Bacteroides intestinalis*	0.25
*Bacteroides massiliensis*	0.25	*Bacteroides massiliensis*	0.26
*Bacteroides ovatus*	0.10	*Bacteroides ovatus*	0.12
*Bacteroides sartorii*	0.19	*Bacteroides rodentium*	0.15
*Bacteroides sp 3.1.40A*	0.24	*Bacteroides sartorii*	0.22
*Bacteroides uniformis*	0.35	*Bacteroides sp 3.1.40A*	0.26
*Bacteroides vulgatus*	0.47	*Bacteroides uniformis*	0.41
*Butyricimonas virosa*	0.41	*Bacteroides vulgatus*	0.53
*Desulfovibrio_u_s*	0.23	*Butyricimonas virosa*	0.48
*Enterococcus_u_s*	0.15	*Desulfovibrio_u_s*	0.26
*Enterococcus faecalis*	0.22	*Enterococcus faecalis*	0.26
*Escherichia coli*	0.52	*Escherichia coli*	0.60
*Parabacteroides_u_s*	0.33	*Parabacteroides_u_s*	0.34
*Parabacteroides distasonis*	0.49	*Parabacteroides distasonis*	0.55
*Parabacteroides goldsteinii*	0.18	*Parabacteroides goldsteinii*	0.21
*Parabacteroides merdae*	0.13	*Parabacteroides merdae*	0.17
*Parabacteroides sp D13*	0.21	*Parabacteroides sp D13*	0.24
*Porphyromonas sp 31.2*	0.15	*Porphyromonas sp 31.2*	0.19
		*Proteus mirabilis*	0.13
**Species decrease associated with infarct**		**Species decrease associated with edema**	
*Bifidobacterium animalis*	−0.21	*Bifidobacterium animalis*	−0.24
*Bifidobacterium bifidum*	−0.13	*Bifidobacterium bifidum*	−0.12
*Bifidobacterium pseudolongum*	−0.14	*Bifidobacterium pseudolongum*	−0.16
*Eubacterium plexicaudatum*	−0.15	*Christensenella_u_s*	−0.13
*Lachnospiraceae bacterium 10-1*	−0.21	*Clostridium saudiense*	−0.08
*Lachnospiraceae bacterium 28-4*	−0.19	*Eubacterium plexicaudatum*	−0.16
*Lachnospiraceae bacterium 3-1*	−0.38	*Imtechella halotolerans*	−0.12
*Lachnospiraceae bacterium A2*	−0.23	*Lachnospiraceae bacterium 10-1*	−0.21
*Lachnospiraceae bacterium A4*	−0.31	*Lachnospiraceae bacterium 28-4*	−0.19
*Lachnospiraceae bacterium COE1*	−0.23	*Lachnospiraceae bacterium 3-1*	−0.41
*Lactobacillus murinus*	−0.37	*Lachnospiraceae bacterium A2*	−0.22
*Oscillibacter sp 1-3*	−0.13	*Lachnospiraceae bacterium A4*	−0.35
*Ruminococcus flavefaciens*	−0.45	*Lachnospiraceae bacterium COE1*	−0.26
*Clostridium clostridioforme*	−0.20	*Lactobacillus animalis*	−0.16
		*Lactobacillus johnsonii*	−0.23
		*Lactobacillus murinus*	−0.42
		*Lactobacillus reuteri*	−0.20
		*Oscillibacter sp 1-3*	−0.11
		*Ruminococcus bromii*	−0.09
		*Ruminococcus flavefaciens*	−0.56
		*Clostridium clostridioforme*	−0.20

P < 0.01 for all bacteria.

**Table 4:** Association of inflammatory markers and bacterial species.

**Feature**	**Coef**	***Q* value**	**Inflammatory marker**	**Coef**	***Q* value**
Aerococcus.urinaeequi	−0.272821	0.008123	Ccl11	−0.20556	1.94E-10
			Ccl12	−0.19400	1.57E-05
			Ccl2	−0.19478	1.43E-06
			Ccl22	−0.19494	2.58E-09
			Ccl24	−0.20162	0.004625
			Ccl4	−0.19439	1.15E-06
			Ccl5	−0.19492	2.35E-09
			Ccl6	−0.19490	1.57E-09
			Cxcl9	−0.18666	0.005476
			Cxcr1	−0.19420	0.002162
			Cxcr5	−0.19052	0.000588
			Il10ra	−0.19083	0.000597
			IL11	−0.19497	2.08E-10
			Il13	−0.19468	9.21E-08
			Il16	−0.19442	1.19E-06
			Il3	−0.21801	8.19E-07
			Il4	−0.19464	4.19E-07
			RGD1561905	−0.20264	0.003346
Aerococcus.viridans	−0.2502272	0.00710756	Ccl11	−0.29201	1.94E-10
			Ccl12	−0.27568	1.57E-05
			Ccl2	−0.27670	1.43E-06
			Ccl22	−0.27693	2.58E-09
			Ccl24	−0.28641	0.004625
			Ccl4	−0.27615	1.15E-06
			Ccl5	−0.27689	2.35E-09
			Ccl6	−0.27687	1.57E-09
			Cxcl9	−0.26517	0.005476
			Cxcr1	−0.27587	0.002162
			Cxcr5	−0.27065	0.000588
			Il10ra	−0.27109	0.000597
			IL11	−0.27697	2.08E-10
			Il13	−0.27655	9.21E-08
			Il16	−0.27619	1.19E-06
			Il3	−0.30971	8.19E-07
			Il4	−0.27650	4.19E-07
			RGD1561905	−0.28786	0.003346
Alistipes.indistinctus	0.49548605	0.00010758	Il17a	0.190118	3.32E-05
Bacteroides.caccae	55294244	2.70E-05	Lfng	0.592807	0.008044
Bacteroides.intestinalis	0.39545256	0.00090266	Ccl19	0.21675248	0.0014087
			Cxcl1	0.23004667	0.00219959
Imtechella.halotolerans	−0.2698021	0.00513494	Tnfrsf11b	−0.3915736	7.93E-16
Parabacteroides.goldsteinii	0.36960675	0.00061701	Il2rb	0.51806	0.00364866
			Il3	0.55259618	2.34E-20
			Il6st	0.52789004	0.00072493
			Tnfsf4	0.51998756	0.00362087
Parabacteroides.sp.D13	0.37087382	0.00010987	Ccl24	0.36028464	0.00462557
Porphyromonas.sp.0.31_2	0.36233409	0.00022155	Ccl24	0.35003715	0.00462557

Q-Value < 0.01 for all bacteria and inflammatory markers.

Negative coefficients for bacteria with negative coefficients for inflammatory markers indicate increase in inflammatory marker.

Positive coefficients for bacteria with positive coefficients for inflammatory markers indicate increase in inflammatory marker.

## 3. Discussion

To our knowledge, we are the first to report on the gut microbial changes with species level resolution in aged male and female rats and to correlate these changes with clinical MRI imaging markers of stroke and inflammatory markers. Following stroke, we found that alpha diversity significantly increased, beta diversity significantly changed, and 5 of the 6 major bacterial phyla were altered. Using machine learning, the top 13 bacterial species that predict whether a sample came from the baseline or post-stroke time point. These bacterial species had independent significant correlations with infarct size, edema size, and CBF. We also identified several species whose interactions with one another were significant in correlating with stroke imaging outcomes. Finally, we found 49 inflammatory markers that correlated with the changes in microbiome from stroke. These changes are representative of a shift from beneficial to pathogenic bacterial species following stroke which results in an increased inflammatory response.

LIF treatment is efficacious in reducing the splenic inflammatory response to stroke (Davis et al., 2020). The gut intestinal stem cells produce LIF for self-renewal of the adult intestinal epithelium (Wang et al., 2020). We hypothesized that LIF would counteract the dysbiosis at the level of the intestinal epithelium. However, this study shows that the presence of LIF has no influence on the changes in the microbiome that occurs after stroke.

**Figure 5:**
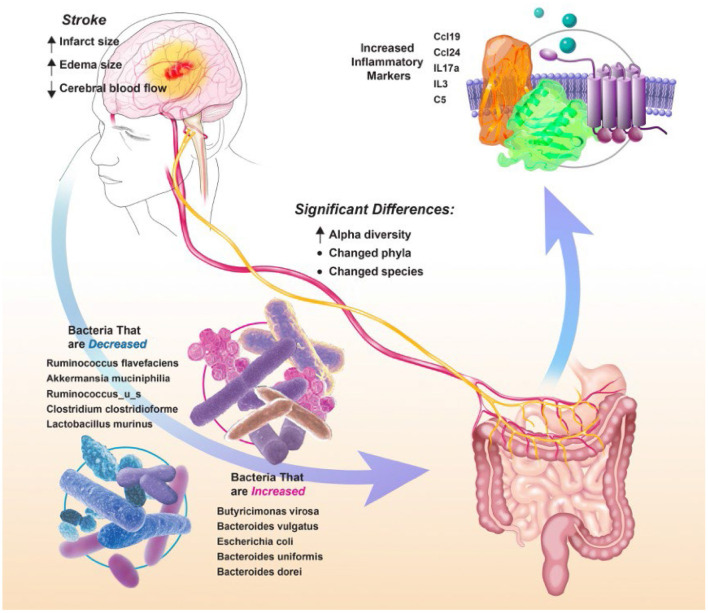
Summary figure depicting changes in gut microbial communities in response to stroke.

Figure 5 summarizes the changes in gut microbial communities in response to stroke. Following stroke there is a significant shift in the gut microbiome, with alterations to 52 major bacterial species. These bacterial fluctuations shift the environment to a more inflammatory state that adversely affect injury. The microbial community dysbiosis is likely due to the increased gut permeability and decreased gut motility in addition to the immunodepression caused by the amplified stress response [increased sympathetic nervous system response and hypothalamic-pituitary-adrenal (HPA) axis response] following stroke (Benakis et al., 2020). Previous groups have reported a decrease in alpha diversity following stroke in a mouse model (Singh et al., 2016) and an increase in a human model (Yin et al., 2015). Our findings are consistent with others who have seen that microbial communities differ before and after stroke based on measures of beta diversity (Park et al., 2020). We did not find any significant differences in the microbiome between males and females. Some groups have found sex differences in the microbiome that are largely attributed to hormone differences (Ahmed and Spence, 2021). It is possible that we did not see these differences because the female rats we used are aged and reproductively senescent.

We saw increases in proteobacteria following stroke. In previous studies, proteobacteria have been associated with increased cognitive impairment following stroke (Ling et al., 2020). Dysbiosis related to metabolic disorders, inflammation, and cancer is often related to an increase in proteobacteria (Shin et al., 2015; Rizzatti et al., 2017). This is possibly due to increased oxygen content in the gut following increases in inflammation, providing an optimal environment for these facultative anaerobes (Rivera-Chavez et al., 2017). We also saw decreases in firmicutes and increases in bacteroidetes species. Decreased firmicutes have also been associated with Alzheimer's disease (Vogt et al., 2017). Actinobacteria were significantly decreased following stroke. Actinobacteria downregulates inflammation by production of IL-4 and IL-13 (Binda et al., 2018).

Many of the bacterial changes were associated with increases in inflammatory markers. The major markers that were increased were CCL19, CCL24, IL-17A, IL-3, and complement factor C5. CCL19 is a chemokine that is commonly upregulated as a result of viral infections (Yan et al., 2019), and attracts dendritic cells and T lymphocytes. CCL19 has previously been found to be upregulated following stroke after damage to the intestinal epithelium (Liu et al., 2017) and has been shown to facilitate T-cell migration to the insult site and microglial activation following stroke (Noor and Wilson, 2012). CCL24 regulates inflammatory and fibrotic activities through its receptor, CCR3 (Segal-Salto et al., 2020). CCR3 is a mediator of neural cell death (Zhang et al., 2016). IL-17A has been shown to be increased following stroke, especially in males (El-Hakim et al., 2021). IL3 is strongly associated with brain volume variation and plays pivotal roles in the expansion and maintenance of the neural progenitor pool and the number of surviving neurons (Luo et al., 2012); our work has previously identified IL3 increased in the spleen with our aged rat model of stroke (Davis et al., 2020). Activated C5 complement components are a part of the cerebral tissue inflammation following ischemia (Costa et al., 2006).

Notably, previous studies have reported age-related changes in the gut microbiome. Spychala et al. (2018) demonstrated that the ratio of firmicutes to bacteroidetes is 9-fold higher in aged mice compared to young mice and that reducing this ratio in aged mice can increase survival following middle cerebral artery occlusion. Lee et al. (2020) showed that transplanting SCFA-producing bacteria prevalent in youthful mice into aged mice following stroke can alleviate poststroke neurological deficits. Finally, Crapser et al. (2016) demonstrated that poststroke infections were more likely to be induced by bacterial translocation following stroke induced gut permeability in aged mice rather than young mice. The present study incorporated aged rats of both sexes in the model of stroke. We found a sharp decrease in the firmicutes to bacteroidetes level rather that an increase. We also found that the SCFA-producing bacteria used in a fecal transplant in the prior study didn't correlate directly with the bacterial species we found to be altered in the current study. The species-level resolution of the current study allows us to pinpoint specific species that could be used for transplantation in future studies to maximize stroke recovery.

This study lays an important foundation upon which precision interventions can be developed to target the gut microbiome in stroke rehabilitation. Future studies should attempt to manipulate the microbiome to change stroke outcomes. This could be achieved through diet interventions, antibiotic therapy, probiotics, or fecal microbiota transplant. For example, a future probiotics study should include the use of *Ruminococcus flavefaciens, Akkermansia muciniphila, and Lactobacillus murinus* as these were deficient in our population. Stroke severity measures from imaging and inflammatory markers could be used as outcomes to compare to the current study. While the present study identified associations of various inflammatory markers with changes in gut microbial composition, it would also be useful to perform mechanistic studies to determine how the microbiota change the expression of these markers and what their downstream effects are. Finally, human studies will be needed to determine whether the microbial changes seen in animals following stroke are similar to the changes seen in animals. Such results can then be used to alter the gut microbiome to favor positive clinical outcomes after stroke.

## 4. Methods

### 4.1. Ethics approval and animals

Details of ethics approval and animals have been reported previously (Messmer et al., 2021). Briefly, aged male and female rats (18-month-old Sprague-Dawley rats (ENVIGO, Indianapolis, IN) were used for all procedures. The aged female rats on average weighed between 245 and 425g, and aged male rats approximately weighed between 505 and 705g. The study was conducted in accordance with the National Institutes of Health Guide for the Care and Use of Laboratory Animals and study protocols were approved by University of Kentucky's (UK) Institutional Animal Care and Use Committee (Protocol number 2020-3516). Reporting in this manuscript follows the recommendations in the ARRIVE guidelines. Animals were housed in an environment-controlled room on a 12-h light and dark cycle (0700–1900) with access to food and water. Per Division of Laboratory Animal Resources (DLAR) cage requirements at UK's vivarium facility, the animals can be paired in one cage if the animal weight is under 650 grams. We typically house two animals (males or females) per cage upon arrival to DLAR. Once the rats are over 650 g, they are then split into a separate cage by themselves. Fecal samples were collected for all animals 22 pMCAO, and 17 tMCAO rats at 24 h before surgery and 72 h post-surgery and for four animals at 30 days post-surgery. The rats underwent MRI at 72 h to measure infarct and edema volumes and CBF then euthanized.

#### 4.1.1. Middle cerebral artery occlusion

Details of the middle cerebral artery occlusion have been reported previously (Messmer et al., 2021). Briefly, 22 of the rats received a permanent Middle Cerebral Artery Occlusion (p-MCAO) and 17 of the rats received a 5-h transient Middle Cerebral Artery Occlusion (5t-MCAO). All animals were induced with oxygen containing 5% isoflurane, then shaved, prepped with Hibiclens (chlorohexidine scrub) prior to 70% EtOH and then a betadine solution. Maintenance isoflurane was maintained at 2.5% in 1 L of O2 and was delivered *via* a nosecone placed in line with the binner tubeQ (gas delivery tube) of the anesthesia circuit. Under bnear sterileQ conditions and with the use of a Zeiss dissectin microscope (Carl Zeiss AG, Gottingen, Germany) at 4–25 magnification, the procedure was performed. First, the skin was opened with a midline vertical incision, and the underlying submandibular gland bluntly dissected in the midline to produce left and right lobes, which were retracted laterally. Division of the omohyoid muscle, then dissection medial to the right sternocleidomastoid (SCM) muscle was used to expose the common carotid artery (CCA), which was separated from the vagus nerve. Elastic hooks (Lone Star Medical Products, Houston, TX, USA) tethered to metal stays on the customized surgery table were used to retract the skin and the SCM muscle. Hand-held electrocautery (Aaron Medical, St. Petersburg, FL, USA) is used to cauterize the superior thyroid artery (STA), a collateral off the ECA, and the occipital artery (OA), a collateral off the ICA. Two 5-0 silk sutures (Surgical Specialties, Reading, PA, USA) were used to ligate the external carotid artery (ECA) as distal as possible to the ECA/ICA bifurcation, and a second tie that was applied just proximal to the first, leaving enough space in between the two ties to cut the artery with micro scissors. At this point, blunt dissection was used to isolate the internal carotid artery (ICA) and its collateral, the pterygopalatine artery. Next, microvascular aneurysm clips (Mizuho, Beverly, MA, USA) were applied to the CCA and the ICA. In the pMCAO procedure, 5-0 PDS II monofilament (Ethicon, Cornelia, GA, USA), was introduced into an arteriotomy hole–produced with a 26-gauge hypodermic needle–in the reflected ECA stump and fed distally into the ICA. At this time, a collar suture at the base of the ECA stump was tightened around the filament, and the ICA clamp was removed. The filament was advanced 20 mm from the carotid bifurcation, with care taken to avoid entrance into the pterygopalatine artery.

For the transient occlusion, the same steps were done as stated with the pMCAO, with the exception that Doccol Corporation silicone rubber-coated monofilaments were used for the occlusion of the middle cerebral artery (MCA). Multiple sized Doccol monofilaments are used in the MCAO surgery depending on the sex and weight of the rat. Two 18-inch length of 5-0 silk suture were used for the ligation of the external carotid artery (ECA) to secure the ECA stump, and the entry point of the monofilament into the ECA/ICA bifurcation. The third 5-0 silk suture was used to secure the monofilament within the ECA. A micro-serrefines arterial clamp (FST, Fine Science Tools, #18055-01) was used to occlude the internal carotid artery (ICA) and common carotid artery (CCA) prior to advancement of the monofilament into the MCA. After 5 h, the filament was gently removed and the collar suture at the base of the ECA stump tightened. The skin was closed with 3-0 nylon suture (Ethicon, Cornelia, GA, USA), anesthesia discontinued, and the animal allowed to recover. Animals used for control underwent a neck dissection and coagulation of the external carotid artery, but no manipulation or occlusion of the common or internal carotid arteries.

### 4.2. Post-surgical fluid management and pain control

Details of the post-surgical fluid management and pain control have been reported previously (Messmer et al., 2021). Briefly, immediately post-operatively the animals received 2 ml of sterile saline (0.9%) subcutaneous. An additional 1 ml of saline was given if extra blood loss occurred during surgery. The animals were injected with sterile filtered PBS pH 7.4 at 6 (for the p-MCAO), 24, 48, and 72 h post-MCAO. The animals were weighed every morning post-MCAO to determine dehydration. Hydration status was checked by pinching up or “tenting” the skin over the nape of the neck. The skin should immediately relax into its normal position. If the skin remains tented longer than normal, the rat was deemed dehydrated, and saline was given. Per DLAR guidelines, rats can receive up to 10 ml at a time and no more than 2 ml at any one location per 6 h. If warranted, additional saline (1–2 ml) will be given in addition to 6, 24, 48, and 72 hr. Also, we added an additional water bottle in each cage to allow more availability to free water for the rats to consume and moistened food was provided on the bottom of the cage to encourage feeding and additional water intake. Post-surgical pain control was managed with carprofen, which is based on weight of the animal. Animal weights are taken prior to surgery (pMCAO) and daily until animals are euthanized at 72 h (post MRI). The animals received a dosage of carprofen 5 mg/kg prior to surgery and every 24 h. for 3 days post-pMCAO until 72 h when they were euthanized (post MRI). Termination of survival criteria include that all animals were weighed and monitored, especially for dehydration and pain, each morning post surgery. This includes specific attention to the animal as a whole, as well as incision sights. If symptoms such as pain, fatigue, loss of energy, excess energy, ruffled hair coat, reluctance to move, failure to groom or feed, hypoactivity, hyperactivity, restlessness, self-trauma, aggressiveness, ataxia, pale mucous membranes, cyanosis, rapid, shallow and/or labored breathing, cachexia, porphyria, soiled anogenital area, inactivity, failure to respond to stimuli, lack of inquisitiveness, vocalization, and/or hunched posture were observed, the research team obtained advice from the vivarium veterinary staff on how best to intervene to alleviate discomfort; if that was not possible the animal was euthanatized. Additional checks were made in the afternoon if there was any rat of concern. The animals were removed from the study if adverse signs persisted despite carprofen and treatment past 24 h. If the signs fail to resolve, the vivarium veterinarian was consulted and decided the time course when such animals were euthanized. Additionally, weight loss >20% (emaciated appearance, rapid weight loss over 2 days) was considered an endpoint. Rapid weight loss was considered >10% a day for 2 days.

### 4.3. Microbiome sequencing

Fecal samples were collected for all animals at 24 h before surgery and 72 h post-surgery and for four animals at 30 days post-surgery. Genomic DNA were extracted from 0.25 g of stool using ZymoBIOMICS™ DNA Mini Kit and shipped to CosmosID for DNA quantification using fluorometer Qubit 3.0. Libraries were constructed and the PCR products were purified using 1.0X speed beads and eluted in 15 μL of nuclease-free water and quantified by PicoGreen fluorometric assay (100X final dilution). The libraries were pooled and loaded onto a high sensitivity chip run on the Caliper LabChipGX (Perkin Elmer, Waltham, MA) for size estimation and sequenced using Illumina NextSeq/HiSeq platform. Unassembled sequencing reads were analyzed by CosmosID bioinformatics platform (CosmosID Inc., Rockville, MD) (Hasan et al., 2014; Lax et al., 2014; Ottesen et al., 2016; Ponnusamy et al., 2016) for microbiome analysis. Heatmaps, stacked bar graphs, and Principal Component Analysis (PCA) plots were generated to visualize the diversity and abundance of each microbial taxa. Alpha- and beta-diversity were calculated to determine the number of species present in a cohort and diversity similarities between groups.

### 4.4. Magnetic resonance imaging

MRI images were acquired on a 7T Bruker Clinscan horizontal bore system (7.0T, 30 cm, 300 Hz) equipped with a triple-axis gradient system (630 mT/m and 6,300 Tm^−1^ s^−1^) with a closed cycle. PCASL (pseudo conintous arterial spin labeling) images were acquired coronally to determine CBF with a fat saturated, double refocused echo planar sequence: TR 4000 ms, TE 26 ms, Matrix 74 x 56, FOV 26 x 19.7 mm, Slice 1.2 mm, Slices 6, 120 Tagged-Untagged Pairs, 10 M_0_ Images, Tagging Plane Offset 12 mm, Bolus duration 1.86 s, Post Labeling Delay 0 s, and Acquisition Time of 10 min. T2 weighted images were acquired coronally with a RARE sequence: TR 6,000 ms, TE 29 ms, Turbo Factor 5, Matrix 190 x 190, FOV 240 x 240 mm, Slice 0.4 mm, Slices 44, and Acquisition Time of 9 min. Male rats were anesthetized with an average of 2.25% isoflurane in oxygen, while female rats were anesthetized with an average of 1.75% isoflurane in oxygen using an MRI compatible CWE Inc. equipment (Ardmore, PA). They were held in place on a Bruker scanning bed with a tooth bar, ear bars, and tape. Body temperature, heart rate, and respiratory rate were continuously monitored throughout the MRI scans (SA Instruments, Inc., Stony Brook, NY). The animal's body temperatures were maintained at 37°C with a water heating system built into the scanning bed. The scanning procedure took ~40–60 min per animal.

The MR images were analyzed by a blinded neuroradiologist who visually identified infarct volume and edema volume. These volumes were counted, and this number was normalized to the number of images counted to provide a per section count. The volume of brain parenchyma demonstrating infarct volume visibly affected was calculated by manual segmentation using ITK-SNAP software (www.itksnap.org, version 3.6) (Yushkevich et al., 2006). The volume of brain parenchyma visibly affected by T2 hyperintensity (edema volume) was calculated in a similar fashion. The data are given as absolute volume in cubic millimeters. The calculation was based on all slices from each MR sequence. Cerebral perfusion values of the area of lesion within the ipsilateral hemisphere, and the equivalent region within the contralateral hemisphere were generated using the quantification as previously described (Lin et al., 2015, 2020).

### 4.5. Biochemical analysis

In following STAIR guidelines, clinically relevant biomarkers were determined in our aged male and female rats (Mehra et al., 2012). Blood was taken from the jugular vein at three different time points: immediately prior to MCAO surgery and 5 min after reperfusion of the MCA in the pMCAO, and 5 h post MCAO procedure in the 5t-MCAO. Blood was immediately placed on ice and centrifuged at 2,000 g for 15 min. Plasma was extracted and stored separately, both pellet and plasma were frozen at −80°C for later analysis. RNA extraction and Amplification followed the methods of Martha et al. (2019). Briefly, total RNA was extracted from the pellet portion *via* a Nucleospin Blood Kit (Macherey-Nagel, Düren, Germany), RNA quantity was estimated using a Qubit 4 Fluorometer (Thermo-Fisher; Waltham, MA), cDNA was synthesized using a RT^2^ PreAMP cDNA synthesis Kit from Qiagen and expression of 84 genes were measured using an ABI StepOne Plus (Germantown, MD) and a RT^2^ Profiler Rat Chemokine and Receptor Array from Qiagen. Delta Delta CT was calculated using the fold change of the gene expression measurement from pre to 3-day.

### 4.6. Statistical analysis

Descriptive microbiome analyses were performed with CosmosID bioinformatics software to generate alpha diversity, beta diversity, and relative abundance data. Alpha diversities amongst groups were compared using Wilcoxon Rank Sum test. Beta diversities amongst groups were compared using PermANOVA. Relative abundance data was compared to measures of stroke severity as determined by imaging (infarct size, edema size, CBF) using general linear models within the MaAsLin 2 R package (Mallick et al., 2021). Random forest was used to determine top bacterial species that were changed following stroke using the randomForest R package (Liaw and Wiener, 2001). All imaging variables in the study were transformed to meet assumptions of normality. The transformation procedures began with Shapiro-Wilks and for measures with *p* < 0.05, the variables were square root transformed. A p-value of 0.05 was set a priori to determine statistical significance.

## Data availability statement

The datasets presented in this study can be found in online repositories. The names of the repository/repositories and accession number(s) can be found below: https://www.ncbi.nlm.nih.gov/sra/, PRJNA883728.

## Ethics statement

The animal study was reviewed and approved by University of Kentucky IACUC Committee.

## Author contributions

TH processed the fecal samples, analyzed the data, and prepared the manuscript. SM performed the stroke surgeries. JF collected the fecal pellets, performed the imaging, and ran the inflammatory analysis. DL interpreted the imaging findings. RC processed the microbiome samples. A-LL and KP oversaw the design and analysis of all experiments. All authors read and approved the final manuscript.

## References

[B1] Ahmed S. Spence J. D. (2021). Sex differences in the intestinal microbiome: interactions with risk factors for atherosclerosis and cardiovascular disease. Biol. Sex Differ. 12, 35. 10.1186/s13293-021-00378-z 34001264 PMC8130173

[B2] Benakis C. Martin-Gallausiaux C. Trezzi J. P. Melton P. Liesz A Wilmes P. (2020). The microbiome-gut-brain axis in acute and chronic brain diseases. Curr. Opin. Neurobiol. 61, 1–9. 10.1016/j.conb.2019.11.009 31812830

[B3] Benjamin E. J. Blaha M. J. Chiuve S. E. Cushman M. Das S. R. Deo R.. (2017). Heart disease and stroke statistics-2017 update: a report from the american heart association. Circulation. 135, e146–e603. 10.1161/CIR.0000000000000491 28122885 PMC5408160

[B4] Binda C. Lopetuso L. R. Rizzatti G. Gibiino G. Cennamo V Gasbarrini A. (2018). Actinobacteria: a relevant minority for the maintenance of gut homeostasis. Dig. Liver Dis. 50, 421–428. 10.1016/j.dld.2018.02.012 29567414

[B5] Carandang R. Seshadri S. Beiser A. Kelly-Hayes M. Kase C. S. Kannel W. B.. (2006). Trends in incidence, lifetime risk, severity, and 30-day mortality of stroke over the past 50 years. JAMA. 296, 2939–2946. 10.1001/jama.296.24.2939 17190894

[B6] Costa C. Zhao L. Shen Y. Su X. Hao L. Colgan S. P.. (2006). Role of complement component C5 in cerebral ischemia/reperfusion injury. Brain Res. 1100, 142–151. 10.1016/j.brainres.2006.05.029 16780818

[B7] Crapser J. Ritzel R. Verma R. Venna V. R. Liu F. Chauhan A.. (2016). Ischemic stroke induces gut permeability and enhances bacterial translocation leading to sepsis in aged mice. Aging (Albany NY). 8, 1049–1063. 10.18632/aging.100952 27115295 PMC4931853

[B8] Davis S. M. Collier L. A. Messmer S. J. Pennypacker R. K. (2020). The poststroke peripheral immune response is differentially regulated by leukemia inhibitory factor in aged male and female rodents. Oxid. Med. Cell. Longev. 2020, 8880244. 10.1155/2020/8880244 33376583 PMC7746465

[B9] El-Hakim Y. Mani K. K. Eldouh A. Pandey S. Grimaldo M. T. Dabney A.. (2021). Sex differences in stroke outcome correspond to rapid and severe changes in gut permeability in adult Sprague-Dawley rats. Biol. Sex Differ. 12, 14. 10.1186/s13293-020-00352-1 33451354 PMC7811247

[B10] Hasan N. A. Young B. A. Minard-Smith A. T. Saeed K. Li H. Heizer E. M.. (2014). Microbial community profiling of human saliva using shotgun metagenomic sequencing. PLoS ONE. 9, e97699. 10.1371/journal.pone.0097699 24846174 PMC4028220

[B11] Lax S. Smith D. P. Hampton-Marcell J. Owens S. M. Handley K. M. Scott N. M.. (2014). Longitudinal analysis of microbial interaction between humans and the indoor environment. Science. 345, 1048–1052. 10.1126/science.1254529 25170151 PMC4337996

[B12] Lee J. d'Aigle J. Atadja L. Quaicoe V. Honarpisheh P. Ganesh B. P.. (2020). Gut Microbiota-derived short-chain fatty acids promote poststroke recovery in aged mice. Circ. Res. 127, 453–465. 10.1161/CIRCRESAHA.119.316448 32354259 PMC7415518

[B13] Leung K. Thuret S (2015). Gut microbiota: a modulator of brain plasticity and cognitive function in ageing. Healthcare (Basel). 3, 898–916. 10.3390/healthcare3040898 27417803 PMC4934620

[B14] Liaw A. Wiener M. (2001). Classification and regression by random forest. Forest. 23, 18–22.

[B15] Lin A. L. Parikh I. Yanckello L. M. White R. S. Hartz A. M. S. Taylor C. E.. (2020). APOE genotype-dependent pharmacogenetic responses to rapamycin for preventing Alzheimer's disease. Neurobiol. Dis. 139, 104834. 10.1016/j.nbd.2020.104834 32173556 PMC7486698

[B16] Lin A. L. Zhang W. Gao X Watts L. (2015). Caloric restriction increases ketone bodies metabolism and preserves blood flow in aging brain. Neurobiol. Aging. 36, 2296–2303. 10.1016/j.neurobiolaging.2015.03.012 25896951 PMC4457572

[B17] Ling Y. Gong T. Zhang J. Gu Q. Gao X. Weng X.. (2020). Gut microbiome signatures are biomarkers for cognitive impairment in patients with ischemic stroke. Front. Aging Neurosci. 12, 511562. 10.3389/fnagi.2020.511562 33192448 PMC7645221

[B18] Liu Y. Luo S. Kou L. Tang C. Huang R. Pei Z. (2017). Ischemic stroke damages the intestinal mucosa and induces alteration of the intestinal lymphocytes and CCL19 mRNA in rats. Neurosci. Lett. 658, 165–170. 10.1016/j.neulet.2017.08.061 28859865

[B19] Longuet-Higgins M. S. (1971). On the Shannon-Weaver index of diversity, in relation to the distribution of species in bird censuses. Theor. Popul. Biol. 2, 271–289. 10.1016/0040-5809(71)90020-7 5162689

[B20] Luo X. J. Li M. Huang L. Nho K. Deng M. Chen Q.. (2012). The interleukin 3 gene (IL3) contributes to human brain volume variation by regulating proliferation and survival of neural progenitors. PLoS ONE. 7, e50375. 10.1371/journal.pone.0050375 23226269 PMC3511536

[B21] Mailing L. J. Allen J. M. Buford T. W. Fields C. J. Woods A. J. (2019). Exercise and the gut microbiome: a review of the evidence, potential mechanisms, and implications for human health. Exerc. Sport Sci. Rev. 47, 75–85. 10.1249/JES.0000000000000183 30883471

[B22] Mallick H. Rahnavard A. McIver L. J. Ma S. Zhang Y. Nguyen L. H.. (2021). Multivariable association discovery in population-scale meta-omics studies. bioRxiv: 2021.2001.2020.427420. 10.1101/2021.01.20.427420 34784344 PMC8714082

[B23] Martha S. R. Cheng Q. Fraser J. F. Gong L. Collier L. A. Davis S. M.. (2019). Expression of cytokines and chemokines as predictors of stroke outcomes in acute ischemic stroke. Front. Neurol. 10, 1391. 10.3389/fneur.2019.01391 32010048 PMC6974670

[B24] Mehra M. Henninger N. Hirsch J. A. Chueh J. Wakhloo A. K. Gounis J. M. (2012). Preclinical acute ischemic stroke modeling. J. Neurointerv. Surg. 4(4): 307–313. 10.1136/neurintsurg-2011-010101 21990535

[B25] Messmer S. J. Salmeron K. E. Frank J. A. McLouth C. J. Lukins D. E. Hammond T. C.. (2021). Extended middle cerebral artery occlusion (mcao) model to mirror stroke patients undergoing thrombectomy. Transl. Stroke Res. 13, 604–615. 10.1007/s12975-021-00936-y 34398389 PMC8847541

[B26] Moy E. Garcia M. C. Bastian B. Rossen L. M. Ingram D. D. Faul M.. (2017). Leading Causes of death in nonmetropolitan and metropolitan areas- United States, 1999-2014. MMWR. Surveill. Summ. 66, 1–8. 10.15585/mmwr.ss6601a1 28081058 PMC5829895

[B27] Noor S. Wilson E. H. (2012). Role of C-C chemokine receptor type 7 and its ligands during neuroinflammation. J. Neuroinflammation. 9, 77. 10.1186/1742-2094-9-77 22533989 PMC3413568

[B28] Ottesen A. Ramachandran P. Reed E. White J. R. Hasan N. Subramanian P.. (2016). Enrichment dynamics of Listeria monocytogenes and the associated microbiome from naturally contaminated ice cream linked to a listeriosis outbreak. BMC Microbiol. 16, 275. 10.1186/s12866-016-0894-1 27852235 PMC5112668

[B29] Park M. J. Pilla R. Panta A. Pandey S. Sarawichitr B. Suchodolski J. (2020). Reproductive senescence and ischemic stroke remodel the gut microbiome and modulate the effects of estrogen treatment in female rats. Transl. Stroke Res. 11, 812–830. 10.1007/s12975-019-00760-5 31845185

[B30] Ponnusamy D. Kozlova E. V. Sha J. Erova T. E. Azar S. R. Fitts E. C.. (2016). Cross-talk among flesh-eating Aeromonas hydrophila strains in mixed infection leading to necrotizing fasciitis. Proc. Natl. Acad. Sci. USA. 113(3): 722–727. 10.1073/pnas.1523817113 26733683 PMC4725515

[B31] Rivera-Chavez F. Lopez C. A. Baumler J. A. (2017). Oxygen as a driver of gut dysbiosis. Free Radic. Biol. Med. 105, 93–101. 10.1016/j.freeradbiomed.2016.09.022 27677568

[B32] Rizzatti G. Lopetuso L. R. Gibiino G. Binda C Gasbarrini A. (2017). Proteobacteria: a common factor in human diseases. Biomed Res. Int. 2017:9351507. 10.1155/2017/9351507 29230419 PMC5688358

[B33] Segal-Salto M. Barashi N. Katav A. Edelshtein V. Aharon A. Hashmueli S.. (2020). A blocking monoclonal antibody to CCL24 alleviates liver fibrosis and inflammation in experimental models of liver damage. JHEP Rep. 2, 100064. 10.1016/j.jhepr.2019.100064 32039405 PMC7005554

[B34] Shin N. R. Whon T. W. Bae W. J. (2015). Proteobacteria: microbial signature of dysbiosis in gut microbiota. Trends Biotechnol. 33, 496–503. 10.1016/j.tibtech.2015.06.011 26210164

[B35] Singh V. Roth S. Llovera G. Sadler R. Garzetti D. Stecher B.. (2016). Microbiota dysbiosis controls the neuroinflammatory response after stroke. J. Neurosci. 36, 7428–7440. 10.1523/JNEUROSCI.1114-16.2016 27413153 PMC6705544

[B36] Spychala M. S. Venna V. R. Jandzinski M. Doran S. J. Durgan D. J. Ganesh B. P.. (2018). Age-related changes in the gut microbiota influence systemic inflammation and stroke outcome. Ann. Neurol. 84, 23–36. 10.1002/ana.25250 29733457 PMC6119509

[B37] Stanley D. Moore R. J. Wong Y. C. H. (2018). An insight into intestinal mucosal microbiota disruption after stroke. Sci. Rep. 8, 568. 10.1038/s41598-017-18904-8 29330443 PMC5766598

[B38] Vogt N. M. Kerby R. L. Dill-McFarland K. A. Harding S. J. Merluzzi A. P. Johnson S. C.. (2017). Gut microbiome alterations in Alzheimer's disease. Sci. Rep. 7, 13537. 10.1038/s41598-017-13601-y 29051531 PMC5648830

[B39] Wang H. Wang J. Zhao Y. Zhang X. Liu J. Zhang C.. (2020). LIF is essential for ISC function and protects against radiation-induced gastrointestinal syndrome. Cell Death Dis. 11, 588. 10.1038/s41419-020-02790-6 32719388 PMC7385639

[B40] Winek K. Meisel A. Dirnagl U. (2016). Gut microbiota impact on stroke outcome: FAD or fact? J. Cereb. Blood Flow Metab. 36, 891–898. 10.1177/0271678X16636890 26945017 PMC4853845

[B41] Yan Y. Chen R. Wang X. Hu K. Huang L. Lu M. (2019). CCL19 and CCR7 expression, signaling pathways, and adjuvant functions in viral infection and prevention. Front Cell Dev. Biol. 7, 212. 10.3389/fcell.2019.00212 31632965 PMC6781769

[B42] Yin J. Liao S. X. He Y. Wang S. Xia G. H. Liu F. T.. (2015). Dysbiosis of gut microbiota with reduced trimethylamine-n-oxide level in patients with large-artery atherosclerotic stroke or transient ischemic attack. J. Am. Heart Assoc. 4, 11. 10.1161/JAHA.115.002699 26597155 PMC4845212

[B43] Yushkevich P. A. Piven J. Hazlett H. C. Smith R. G. Ho S. Gee J. C. (2006). User-guided 3D active contour segmentation of anatomical structures: significantly improved efficiency and reliability. Neuroimage. 31, 1116–1128. 10.1016/j.neuroimage.2006.01.015 16545965

[B44] Zhang J. Wang H. Sherbini O. Ling-Lin Pai E. Kang S. U. Kwon J. S.. (2016). High-content genome-wide RNAi screen reveals CCR3 as a key mediator of neuronal cell death. eNeuro. 3, ENEURO.0185-16.2016. 10.1523/ENEURO.0185-16.2016 27822494 PMC5075945

