# Supplementary material for: Gut microbial dysbiosis correlates with stroke severity markers in aged rats
Source: Front Stroke. 2022 Dec 21;1:1026066. doi: 10.3389/fstro.2022.1026066 (PMC9945937; doi:10.3389/fstro.2022.1026066)
Supplement: Supplementary file 1 [file Table_1.DOCX]

**Supplementary Table 1**. PermANOVA analysis to measure significance differences between groups using Bray-Curtis Index.

| Cohort1 | Cohort2 | Sample Size | Statistic | P-Value |
| --- | --- | --- | --- | --- |
| Baseline | Stroke | 74 | 6.178096083 | **0.001** |
| Permanent | Temporary | 37 | 0.88984527 | 0.583 |
| LIF | PBS | 37 | 0.682004965 | 0.865 |
| Male | Female | 32 | 3.784863342 | 0.339 |

**Supplementary Table 2.** A) Statistical Analysis reveals significant changes in major phyla, B) Models predicting infarct and edema by major phyla

A)

| Phylum | Change in Relative Abundance | 95% CI | p-value |
| --- | --- | --- | --- |
| Proteobacteria | 3.1819 | (2.2620, 4.8007) | **<0.0001** |
| Firmicutes | 0.7245 | (0.6003, 0.8604) | **0.0003** |
| Bacteroidetes | 3.9310 | (2.6026, 5.9388) | **<0.0001** |
| Verrucomicrobia | 0.5530 | (0.3764, 0.8124) | **0.0035** |
| Deferribacteres | 1.3935 | (0.4078, 4.7610) | 0.5877 |
| Actinobacteria | 0.5822 | (0.4356, 0.7780) | **0.0005** |
| FB Ratio | 0.1692 | (0.0977, 0.2930) | **<0.0001** |

B)

| **Predicting Infarct** | **RSquare 0.3866** | | | | |
| --- | --- | --- | --- | --- | --- |
| Parameter | Estimate | DF | SS | F Ratio | P-value |
| Intercept | -42.46 | 1 | 0 | 0 | 1 |
| Firmicutes | 34.55 | 1 | 132.17 | 6.93 | 0.0129 |
| Actinobacteria | 37.44 | 1 | 114.56 | 6.01 | 0.0199 |
| Proteobacteria | 34.04 | 1 | 143.03 | 7.50 | 0.01 |
| Bacteroides | 41.12 | 1 | 161.67 | 8.48 | 0.0065 |

| **Predicting Edema** | **RSquare 0.6022** | | | | |
| --- | --- | --- | --- | --- | --- |
| Parameter | Estimate | DF | SS | F Ratio | P-value |
| Intercept | -47.09 | 1 | 0 | 0 | 1 |
| Proteobacteria | 41.22 | 1 | 180.55 | 13.16 | 0.0010 |
| Bacteroides | 46.49 | 1 | 200.49 | 14.62 | 0.0006 |
| Verrucomicrobia | 18.83 | 1 | 41.16 | 3.00 | 0.0932 |
| Firmicutes | 35.12 | 1 | 124.48 | 9.08 | 0.0051 |
| Actinobacteria | 46.14 | 1 | 156.44 | 11.41 | 0.0020 |

**Supplementary Table 3**. Taxa which were increased (red) and decreased (green) following stroke.

| **Phylum** | **Class** | **Order** | **Family** | **Genus** | **Species** |
| --- | --- | --- | --- | --- | --- |
| Actinobacteria | Actinobacteria | Bifidobacteriales | Bifidobacteriaceae | Bifidobacterium | Bifidobacterium animalis |
|  |  |  |  |  | Bifidobacterium bifidum |
|  |  |  |  |  | Bifidobacterium choerinum |
|  |  |  |  |  | Bifidobacterium pseudocatenulatum |
|  |  |  |  |  | Bifidobacterium pseudolongum |
|  |  |  |  |  | Bifidobacterium pullorum |
|  |  |  |  |  | Bifidobacterium sp. AGR2158 |
|  |  | Corynebacteriales | Corynebacteriaceae | Corynebacterium | Corynebacterium ammoniagenes |
|  |  |  |  |  | Corynebacterium casei |
|  |  |  |  |  | Corynebacterium glutamicum |
|  |  |  |  |  | Corynebacterium sp. HFH0082 |
|  |  | Micrococcales | Micrococcaceae | Rothia | Rothia_u_s |
|  | Coriobacteriia | Coriobacteriales | Coriobacteriaceae | Coriobacteriaceae_u_g | Coriobacteriaceae bacterium 68-1-3 |
|  |  | Eggerthellales | Eggerthellaceae | Adlercreutzia | Adlercreutzia equolifaciens |
|  |  |  |  | Enterorhabdus | Enterorhabdus caecimuris |
|  |  |  |  |  | Enterorhabdus mucosicola |
|  |  |  |  |  | Enterorhabdus_u_s |
| Bacteroidetes | Bacteroidia | Bacteroidales | Bacteroidaceae | Bacteroides | Bacteroides acidifaciens |
|  |  |  |  |  | Bacteroides caccae |
|  |  |  |  |  | Bacteroides dorei |
|  |  |  |  |  | Bacteroides eggerthii |
|  |  |  |  |  | Bacteroides faecichinchillae |
|  |  |  |  |  | Bacteroides finegoldii |
|  |  |  |  |  | Bacteroides fragilis |
|  |  |  |  |  | Bacteroides intestinalis |
|  |  |  |  |  | Bacteroides massiliensis |
|  |  |  |  |  | Bacteroides ovatus |
|  |  |  |  |  | Bacteroides rodentium |
|  |  |  |  |  | Bacteroides sartorii |
|  |  |  |  |  | Bacteroides sp. 2_1_33B |
|  |  |  |  |  | Bacteroides sp. 3_1_40A |
|  |  |  |  |  | Bacteroides sp. 4_1_36 |
|  |  |  |  |  | Bacteroides sp. 4_3_47FAA |
|  |  |  |  |  | Bacteroides sp. D20 |
|  |  |  |  |  | Bacteroides stercoris |
|  |  |  |  |  | Bacteroides thetaiotaomicron |
|  |  |  |  |  | Bacteroides uniformis |
|  |  |  |  |  | Bacteroides vulgatus |
|  |  |  |  |  | Bacteroides xylanisolvens |
|  |  |  |  |  | Bacteroides_u_s |
|  |  |  | Bacteroidales_u_f | Bacteroidales_u_g | Bacteroidales_u_s |
|  |  |  | Barnesiellaceae | Barnesiella | Barnesiella intestinihominis |
|  |  |  | Odoribacteraceae | Butyricimonas | Butyricimonas virosa |
|  |  |  |  |  | Butyricimonas_u_s |
|  |  |  | Porphyromonadaceae | Porphyromonas | Porphyromonas sp. 31_2 |
|  |  |  | Prevotellaceae | Paraprevotella | Paraprevotella clara |
|  |  |  |  |  | Paraprevotella xylaniphila |
|  |  |  |  |  | Paraprevotella_u_s |
|  |  |  | Rikenellaceae | Alistipes | Alistipes finegoldii |
|  |  |  |  |  | Alistipes indistinctus |
|  |  |  |  |  | Alistipes onderdonkii |
|  |  |  |  |  | Alistipes putredinis |
|  |  |  |  |  | Alistipes shahii |
|  |  |  |  |  | Alistipes sp. HGB5 |
|  |  |  |  |  | Alistipes timonensis |
|  |  |  |  |  | Alistipes_u_s |
|  |  |  | Tannerellaceae | Parabacteroides | Parabacteroides distasonis |
|  |  |  |  |  | Parabacteroides goldsteinii |
|  |  |  |  |  | Parabacteroides johnsonii |
|  |  |  |  |  | Parabacteroides merdae |
|  |  |  |  |  | Parabacteroides sp. 20_3 |
|  |  |  |  |  | Parabacteroides sp. D13 |
|  |  |  |  |  | Parabacteroides_u_s |
|  | Flavobacteriia | Flavobacteriales | Flavobacteriaceae | Imtechella | Imtechella halotolerans |
| Deferribacteres | Deferribacteres | Deferribacterales | Deferribacteraceae | Mucispirillum | Mucispirillum schaedleri |
| Firmicutes | Bacilli | Bacillales | Staphylococcaceae | Jeotgalicoccus | Jeotgalicoccus_u_s |
|  |  |  |  | Staphylococcus | Staphylococcus aureus |
|  |  |  |  |  | Staphylococcus lentus |
|  |  |  |  |  | Staphylococcus_u_s |
|  |  | Lactobacillales | Aerococcaceae | Aerococcus | Aerococcus urinaeequi |
|  |  |  |  |  | Aerococcus viridans |
|  |  |  |  |  | Aerococcus_u_s |
|  |  |  | Enterococcaceae | Enterococcus | Enterococcus avium |
|  |  |  |  |  | Enterococcus faecalis |
|  |  |  |  |  | Enterococcus faecium |
|  |  |  |  |  | Enterococcus gallinarum |
|  |  |  |  |  | Enterococcus saccharolyticus |
|  |  |  |  |  | Enterococcus sp. HSIEG1 |
|  |  |  |  |  | Enterococcus_u_s |
|  |  |  | Lactobacillaceae | Lactobacillus | Lactobacillus acidophilus |
|  |  |  |  |  | Lactobacillus amylovorus |
|  |  |  |  |  | Lactobacillus animalis |
|  |  |  |  |  | Lactobacillus johnsonii |
|  |  |  |  |  | Lactobacillus murinus |
|  |  |  |  |  | Lactobacillus reuteri |
|  |  |  |  |  | Lactobacillus salivarius |
|  |  |  |  |  | Lactobacillus sp. ASF360 |
|  |  |  |  |  | Lactobacillus vaginalis |
|  |  |  |  |  | Lactobacillus_u_s |
|  |  |  |  | Pediococcus | Pediococcus pentosaceus |
|  |  |  | Leuconostocaceae | Weissella | Weissella cibaria |
|  |  |  |  |  | Weissella hellenica |
|  |  |  |  |  | Weissella_u_s |
|  |  |  | Streptococcaceae | Lactococcus | Lactococcus garvieae |
|  |  |  |  |  | Lactococcus_u_s |
|  |  |  |  | Streptococcus | Streptococcus agalactiae |
|  |  |  |  |  | Streptococcus oralis |
|  |  |  |  |  | Streptococcus_u_s |
|  | Clostridia | Clostridiales | Christensenellaceae | Christensenella | Christensenella timonensis |
|  |  |  |  |  | Christensenella_u_s |
|  |  |  | Clostridiaceae | Clostridiaceae_u_g | Clostridiaceae_u_s |
|  |  |  |  | Clostridium | Clostridium celatum |
|  |  |  |  |  | Clostridium saudiense |
|  |  |  |  |  | Clostridium sp. ASF502 |
|  |  |  |  |  | Clostridium sp. ATCC 29733 |
|  |  |  |  |  | Clostridium sp. ATCC BAA-442 |
|  |  |  |  |  | Clostridium_u_s |
|  |  |  |  | Hungatella | Hungatella hathewayi |
|  |  |  | Clostridiales_u_f | Clostridiales_u_g | Clostridiales bacterium VE202-06 |
|  |  |  |  |  | Clostridiales_u_s |
|  |  |  |  | Flavonifractor | Flavonifractor plautii |
|  |  |  |  | Intestinimonas | Intestinimonas butyriciproducens |
|  |  |  |  | Pseudoflavonifractor | Pseudoflavonifractor capillosus |
|  |  |  | Eubacteriaceae | Eubacterium | Eubacterium plexicaudatum |
|  |  |  |  |  | Eubacterium sp. 14-2 |
|  |  |  | Lachnospiraceae | Blautia | Blautia producta |
|  |  |  |  | Dorea | Dorea sp. 5-2 |
|  |  |  |  | Lachnoanaerobaculum | Lachnoanaerobaculum sp. OBRC5-5 |
|  |  |  |  | Lachnoclostridium | [Clostridium] clostridioforme |
|  |  |  |  | Lachnospiraceae_u_g | Lachnospiraceae bacterium 10-1 |
|  |  |  |  |  | Lachnospiraceae bacterium 28-4 |
|  |  |  |  |  | Lachnospiraceae bacterium 3-1 |
|  |  |  |  |  | Lachnospiraceae bacterium 3-2 |
|  |  |  |  |  | Lachnospiraceae bacterium 7_1_58FAA |
|  |  |  |  |  | Lachnospiraceae bacterium A2 |
|  |  |  |  |  | Lachnospiraceae bacterium A4 |
|  |  |  |  |  | Lachnospiraceae bacterium COE1 |
|  |  |  |  |  | Lachnospiraceae bacterium M18-1 |
|  |  |  |  |  | Lachnospiraceae_u_s |
|  |  |  |  | Roseburia | Roseburia intestinalis |
|  |  |  | Oscillospiraceae | Oscillibacter | Oscillibacter sp. 1-3 |
|  |  |  |  |  | Oscillibacter_u_s |
|  |  |  |  | Oscillospiraceae_u_g | Oscillospiraceae_u_s |
|  |  |  | Peptostreptococcaceae | Clostridioides | Clostridioides difficile |
|  |  |  |  | Peptostreptococcaceae_u_g | Peptostreptococcaceae bacterium VA2 |
|  |  |  |  |  | Peptostreptococcaceae_u_s |
|  |  |  |  | Romboutsia | [Clostridium] dakarense |
|  |  |  |  | Terrisporobacter | Terrisporobacter_u_s |
|  |  |  | Ruminococcaceae | Anaerotruncus | Anaerotruncus sp. G3(2012) |
|  |  |  |  | Faecalibacterium | Faecalibacterium prausnitzii |
|  |  |  |  | Ruminiclostridium | [Eubacterium] siraeum |
|  |  |  |  | Ruminococcaceae_u_g | Ruminococcaceae bacterium D16 |
|  |  |  |  |  | Ruminococcaceae_u_s |
|  |  |  |  | Ruminococcus | Ruminococcus bromii |
|  |  |  |  |  | Ruminococcus flavefaciens |
|  |  |  |  |  | Ruminococcus_u_s |
|  |  |  |  | Subdoligranulum | Subdoligranulum sp. 4_3_54A2FAA |
|  |  |  |  |  | Subdoligranulum_u_s |
|  | Erysipelotrichia | Erysipelotrichales | Erysipelotrichaceae | Allobaculum | Allobaculum stercoricanis |
|  |  |  |  | Erysipelotrichaceae_u_g | Erysipelotrichaceae bacterium 6_1_45 |
|  |  |  |  | Turicibacter | Turicibacter sanguinis |
|  |  |  |  |  | Turicibacter_u_s |
|  | Firmicutes_u_c | Firmicutes_u_o | Firmicutes_u_f | Firmicutes_u_g | Firmicutes bacterium ASF500 |
|  |  |  |  |  | Firmicutes bacterium M10-2 |
|  | Negativicutes | Acidaminococcales | Acidaminococcaceae | Acidaminococcus | Acidaminococcus_u_s |
|  |  | Selenomonadales | Selenomonadaceae | Megamonas | Megamonas_u_s |
| Proteobacteria | Betaproteobacteria | Burkholderiales | Burkholderiales_u_f | Burkholderiales_u_g | Burkholderiales bacterium 1_1_47 |
|  |  |  |  |  | Burkholderiales_u_s |
|  |  |  | Sutterellaceae | Parasutterella | Parasutterella excrementihominis |
|  |  |  |  | Sutterella | Sutterella wadsworthensis |
|  | Deltaproteobacteria | Desulfovibrionales | Desulfovibrionaceae | Bilophila | Bilophila_u_s |
|  |  |  |  | Desulfovibrio | Desulfovibrio desulfuricans |
|  |  |  |  |  | Desulfovibrio sp. Dsv1 |
|  |  |  |  |  | Desulfovibrio_u_s |
|  | Gammaproteobacteria | Enterobacterales | Enterobacteriaceae | Enterobacter | Enterobacter cancerogenus |
|  |  |  |  |  | Enterobacter cloacae |
|  |  |  |  |  | Enterobacter cloacae complex |
|  |  |  |  |  | Enterobacter hormaechei |
|  |  |  |  |  | Enterobacter sp. MGH 38 |
|  |  |  |  | Escherichia | Escherichia coli |
|  |  |  |  |  | Escherichia_u_s |
|  |  |  |  | Klebsiella | Klebsiella_u_s |
|  |  |  |  | Shigella | Shigella flexneri |
|  |  |  |  |  | Shigella sonnei |
|  |  |  |  |  | Shigella_u_s |
|  |  |  | Morganellaceae | Morganella | Morganella morganii |
|  |  |  |  |  | Morganella sp. EGD-HP17 |
|  |  |  |  |  | Morganella_u_s |
|  |  |  |  | Proteus | Proteus mirabilis |
|  |  |  |  |  | Proteus_u_s |
|  |  | Pseudomonadales | Moraxellaceae | Psychrobacter | Psychrobacter phenylpyruvicus |
|  |  |  |  |  | Psychrobacter_u_s |
| Verrucomicrobia | Verrucomicrobiae | Verrucomicrobiales | Akkermansiaceae | Akkermansia | Akkermansia muciniphila |

**Supplementary Table 4.** FSA Analysis testing to test significant interactions in predicting infarct, edema, and CBF.

| **Predicting Infarct** | **RSquare 0.6206** | | | | |
| --- | --- | --- | --- | --- | --- |
| Parameter | Estimate | Std Error | T Ratio |  | P-value |
| Intercept | 8.45 | 0.82 | 10.33 |  | <0.0001 |
| Lachnospiraceae bacterium A2 | -1856.40 | 386.89 | -4.80 |  | <0.0001 |
| Lactobacillus murinus | -58.95 | 22.06 | -2.67 |  | 0.0121 |
| Ruminococcus_u_s | 177.34 | 462.94 | 0.38 |  | 0.7044 |
| Lactobacillus murinus* Lachnospiraceae bacterium A2 | 44708.92 | 15163.12 | 2.95 |  | 0.0061 |
| Ruminococcus_u_s* Lachnospiraceae bacterium A2 | -602368.5 | 220226.4 | -2.74 |  | 0.0104 |
| Ruminococcus_u_s* Lactobacillus murinus | 41430.66 | 11426.45 | 3.63 |  | 0.0011 |

| **Predicting Edema** | **RSquare 0.6454** | | | | |
| --- | --- | --- | --- | --- | --- |
| Parameter | Estimate | Std Error | T Ratio |  | P-value |
| Intercept | 11.69 | 0.83 | 14.18 |  | <0.0001 |
| Lachnospiraceae bacterium A4 | -1512.88 | 387.28 | -3.91 |  | 0.0005 |
| Lactobacillus murinus | -71.11 | 23.86 | -2.98 |  | 0.0056 |
| Ruminococcus_u_s | -400.57 | -400.57 | -0.88 |  | 0.3834 |
| Lactobacillus murinus* Lachnospiraceae bacterium A4 | 45536.34 | 16933.4 | 2.69 |  | 0.0114 |
| Lactobacillus murinus* Ruminococcus_u_s | 25866.25 | 8928.12 | 2.90 |  | 0.0069 |

| **Predicting CBF** | **RSquare 0.8093** | | | | |
| --- | --- | --- | --- | --- | --- |
| Parameter | Estimate | Std Error | T Ratio |  | P-value |
| Intercept | 2.76 | 0.16 | 17.13 |  | <0.0001 |
| Adlercreutzia equolifaciens | -17.71 | 3.54 | -5.00 |  | <0.0001 |
| Desulfovibrio desulfuricans | -680.87 | 303.69 | -2.24 |  | 0.0331 |
| Lactobacillus acidophilus | -18.82 | 4.60 | -4.09 |  | 0.0003 |
| Parabacteroides sp. D13 | -37.07 | 6.23 | -5.95 |  | <0.0001 |
| Desulfovibrio desulfuricans* Adlercreutzia equolifaciens | 131344.78 | 23952.7 | 5.48 |  | <0.0001 |
| Parabacteroides sp. D13* Adlercreutzia equolifaciens | -1225.13 | 232.89 | -5.26 |  | <0.0001 |
| Parabacteroides sp. D13* Desulfovibrio desulfuricans | 46301.94 | 19481.43 | 2.38 |  | 0.0245 |
| Parabacteroides sp. D13* Lactobacillus acidophilus | -890.06 | 398.37 | -2.23 |  | 0.0336 |

**Supplementary Table 5**. Inflammatory Marker Associations

| Inflammatory Marker | feature | coef | stderr | qval |
| --- | --- | --- | --- | --- |
| Aimp1 | Lactobacillus.sp..ASF360 | -0.5176314 | 0.08840525 | 0.04185764 |
|  | Parabacteroides.goldsteinii | 0.48399257 | 0.08945788 | 0.04409221 |
| Ccl11 | Aerococcus.urinaeequi | -0.2055613 | 3.02E-05 | 1.94E-10 |
|  | Aerococcus.viridans | -0.2920137 | 4.29E-05 | 1.94E-10 |
|  | Enterococcus.avium | 0.2127899 | 0.03764904 | 0.01429911 |
|  | Intestinimonas.butyriciproducens | -0.1447379 | 2.13E-05 | 1.94E-10 |
|  | Lactobacillus.sp..ASF360 | -0.5563291 | 0.07859435 | 0.00438146 |
|  | Rothia_u_s | -0.1003455 | 1.47E-05 | 1.94E-10 |
| Ccl12 | Aerococcus.urinaeequi | -0.194066 | 0.00316641 | 1.57E-05 |
|  | Aerococcus.viridans | -0.2756839 | 0.00449811 | 1.57E-05 |
|  | Enterococcus.avium | 0.20569703 | 0.03149521 | 0.00404166 |
|  | Lactobacillus.sp..ASF360 | -0.5393555 | 0.07032229 | 0.00163928 |
|  | Parabacteroides.goldsteinii | 0.4622389 | 0.10361534 | 0.03899742 |
|  | Rothia_u_s | -0.0947341 | 0.0015457 | 1.57E-05 |
| Ccl19 | Bacteroides.intestinalis | 0.21675248 | 0.01467556 | 0.0014087 |
|  | Christensenella_u_s | -0.3512259 | 0.06027937 | 0.03728309 |
|  | Escherichia.coli | 0.98364203 | 0.11364189 | 0.01148311 |
|  | Firmicutes.bacterium.ASF500 | -0.5711 | 0.10127128 | 0.02547683 |
|  | Lachnospiraceae.bacterium.3.1 | -0.6003293 | 0.10340114 | 0.02547683 |
|  | Oscillibacter.sp..1.3 | -0.4116514 | 0.0738624 | 0.02547683 |
|  | Proteus.mirabilis | 0.41021879 | 0.07965629 | 0.0374422 |
|  | Ruminococcus.flavefaciens | -0.7961519 | 0.13105375 | 0.02547683 |
| Ccl2 | Aerococcus.urinaeequi | -0.1947833 | 0.00175363 | 1.43E-06 |
|  | Aerococcus.viridans | -0.2767028 | 0.00249116 | 1.43E-06 |
|  | Enterococcus.avium | 0.20487031 | 0.03216172 | 0.00470742 |
|  | Lactobacillus.sp..ASF360 | -0.5370347 | 0.07250851 | 0.00206344 |
|  | Parabacteroides.goldsteinii | 0.45987351 | 0.10492274 | 0.04250008 |
|  | Rothia_u_s | -0.0950842 | 0.00085604 | 1.43E-06 |
| Ccl20 | Akkermansia.muciniphila | 0.55013115 | 0.02812086 | 0.0258554 |
| Ccl22 | Aerococcus.urinaeequi | -0.1949475 | 0.00036149 | 2.58E-09 |
|  | Aerococcus.viridans | -0.2769361 | 0.00051352 | 2.58E-09 |
|  | Enterococcus.avium | 0.20356816 | 0.03317916 | 0.00606937 |
|  | Lactobacillus.sp..ASF360 | -0.5359237 | 0.07352888 | 0.00231125 |
|  | Parabacteroides.goldsteinii | 0.46100688 | 0.10429919 | 0.04044564 |
|  | Rothia_u_s | -0.0951644 | 0.00017646 | 2.58E-09 |
| Ccl24 | Aerococcus.urinaeequi | -0.2016234 | 0.01131589 | 0.00462557 |
|  | Aerococcus.viridans | -0.2864196 | 0.01607499 | 0.00462557 |
|  | Bacteroides.fragilis | 0.09925927 | 0.00552128 | 0.00462557 |
|  | Bifidobacterium.bifidum | -0.0366109 | 0.00204364 | 0.00462557 |
|  | Enterococcus.avium | 0.22153327 | 0.02957968 | 0.00462557 |
|  | Lactobacillus.sp..ASF360 | -0.5920759 | 0.03425105 | 0.00012409 |
|  | Parabacteroides.sp..D13 | 0.36028464 | 0.0519538 | 0.00462557 |
|  | Porphyromonas.sp..31_2 | 0.35003715 | 0.05151981 | 0.00462557 |
|  | Rothia_u_s | -0.0984232 | 0.0055239 | 0.00462557 |
| Ccl3 | Aerococcus.urinaeequi | -0.1765955 | 0.02161083 | 0.03235321 |
|  | Aerococcus.viridans | -0.2508658 | 0.03069965 | 0.03235321 |
|  | Enterococcus.avium | 0.19076089 | 0.04160564 | 0.03547143 |
|  | Lactobacillus.sp..ASF360 | -0.5396346 | 0.07005413 | 0.00624261 |
|  | Parabacteroides.goldsteinii | 0.47108973 | 0.0987267 | 0.03331638 |
|  | Rothia_u_s | -0.0862058 | 0.01054941 | 0.03235321 |
| Ccl4 | Aerococcus.urinaeequi | -0.1943963 | 0.0016556 | 1.15E-06 |
|  | Aerococcus.viridans | -0.2761531 | 0.0023519 | 1.15E-06 |
|  | Enterococcus.avium | 0.20260383 | 0.03390896 | 0.00724995 |
|  | Lactobacillus.sp..ASF360 | -0.5368243 | 0.07270308 | 0.0021088 |
|  | Parabacteroides.goldsteinii | 0.46377242 | 0.1027552 | 0.0357345 |
|  | Rothia_u_s | -0.0948953 | 0.00080819 | 1.15E-06 |
| Ccl5 | Aerococcus.urinaeequi | -0.19492 | 0.00035154 | 2.35E-09 |
|  | Aerococcus.viridans | -0.276897 | 0.00049938 | 2.35E-09 |
|  | Enterococcus.avium | 0.20300111 | 0.03361064 | 0.00686864 |
|  | Lactobacillus.sp..ASF360 | -0.535723 | 0.07371145 | 0.00240153 |
|  | Parabacteroides.goldsteinii | 0.46193616 | 0.10378397 | 0.03952765 |
|  | Rothia_u_s | -0.0951509 | 0.0001716 | 2.35E-09 |
| Ccl6 | Aerococcus.urinaeequi | -0.1949019 | 0.0003201 | 1.57E-09 |
|  | Aerococcus.viridans | -0.2768714 | 0.00045473 | 1.57E-09 |
|  | Enterococcus.avium | 0.2034532 | 0.03326719 | 0.00614521 |
|  | Lactobacillus.sp..ASF360 | -0.5360972 | 0.07337066 | 0.00225035 |
|  | Parabacteroides.goldsteinii | 0.46154141 | 0.10400327 | 0.03913961 |
|  | Rothia_u_s | -0.0951421 | 0.00015626 | 1.57E-09 |
| Ccl7 | Enterococcus.avium | 0.2269836 | 0.02317275 | 0.0033076 |
|  | Proteus.mirabilis | 0.40028778 | 0.06198216 | 0.02099886 |
| Ccl9 | Akkermansia.muciniphila | -0.4483565 | 0.07146225 | 0.02585543 |
| Ccr1 | Clostridiales_u_s | -0.6232898 | 0.08526963 | 0.02809096 |
|  | Firmicutes.bacterium.ASF500 | -0.5451876 | 0.09352121 | 0.02420724 |
|  | Oscillibacter.sp..1.3 | -0.3809227 | 0.06356631 | 0.02420724 |
| Ccr2 | Aerococcus.urinaeequi | -0.175319 | 0.02536539 | 0.04027451 |
|  | Aerococcus.viridans | -0.2490524 | 0.03603324 | 0.04027451 |
|  | Enterococcus.avium | 0.22151833 | 0.01240135 | 1.24E-05 |
|  | Firmicutes.bacterium.ASF500 | -0.490113 | 0.09534454 | 0.04027451 |
|  | Lactobacillus.sp..ASF360 | -0.5312128 | 0.0776846 | 0.00722588 |
|  | Rothia_u_s | -0.0855826 | 0.01238221 | 0.04027451 |
| Ccr3 | Enterococcus.avium | 0.21605877 | 0.0211112 | 0.00077095 |
|  | Firmicutes.bacterium.ASF500 | -0.5048943 | 0.07011542 | 0.03733616 |
|  | Firmicutes.bacterium.M10.2 | -0.2292557 | 0.02868175 | 0.03733616 |
|  | X.Clostridium..clostridioforme | -0.3376655 | 0.07057603 | 0.03733616 |
| Csf1 | Clostridiales_u_s | -0.5922489 | 0.09276224 | 0.0467224 |
|  | Enterococcus.avium | 0.20603125 | 0.03088165 | 0.01089215 |
|  | Firmicutes.bacterium.ASF500 | -0.5320102 | 0.07971418 | 0.02165814 |
|  | Firmicutes.bacterium.M10.2 | -0.2369883 | 0.01741289 | 0.01089215 |
|  | Oscillibacter.sp..1.3 | -0.3148833 | 0.04888389 | 0.0467224 |
|  | X.Clostridium..clostridioforme | -0.3555341 | 0.05858887 | 0.01089215 |
| Cx3cl1 | Lactobacillus.vaginalis | 0.1277695 | 0.01074746 | 0.00025632 |
|  | Parabacteroides.sp..20_3 | -0.1036426 | 0.01252312 | 0.03124284 |
|  | Ruminococcus.bromii | -0.1486714 | 0.01796394 | 0.03124284 |
|  | Turicibacter_u_s | -0.558161 | 0.06744252 | 0.03124284 |
|  | Enterococcus.avium | 0.21092958 | 0.02626396 | 0.00437503 |
|  | Lactobacillus.sp..ASF360 | -0.5364668 | 0.07303211 | 0.00437503 |
|  | Parabacteroides.goldsteinii | 0.49042248 | 0.08503199 | 0.01949393 |
| Cxcl1 | Bacteroides.intestinalis | 0.23004667 | 0.01482397 | 0.00219959 |
| Cxcl11 | Peptostreptococcaceae_u_s | -0.4865998 | 0.06644799 | 0.01778353 |
|  | Pseudoflavonifractor.capillosus | 0.21862463 | 0.03142318 | 0.0126942 |
| Cxcl12 | Lactobacillus.vaginalis | 0.12065556 | 0.01829574 | 0.01856474 |
| Cxcl2 | Bacteroides.fragilis | 0.23369716 | 0.00446897 | 8.44E-05 |
|  | Bacteroides.rodentium | 0.49586934 | 0.0864276 | 0.0116428 |
|  | Parabacteroides.sp..D13 | 0.46764386 | 0.05556739 | 0.0116428 |
|  | Porphyromonas.sp..31_2 | 0.44585835 | 0.07522601 | 0.0116428 |
| Cxcl6 | Lactobacillus.vaginalis | 0.12956243 | 0.00726643 | 1.20E-05 |
|  | Parabacteroides.sp..20_3 | -0.1041326 | 0.00805052 | 0.00546661 |
|  | Ruminococcus.bromii | -0.1493743 | 0.01154816 | 0.00546661 |
|  | Turicibacter_u_s | -0.5608 | 0.04335559 | 0.00546661 |
| Cxcl9 | Aerococcus.urinaeequi | -0.1866675 | 0.01528793 | 0.00547613 |
|  | Aerococcus.viridans | -0.2651738 | 0.02171754 | 0.00547613 |
|  | Enterococcus.avium | 0.21588536 | 0.02139339 | 0.00087284 |
|  | Lactobacillus.sp..ASF360 | -0.5381751 | 0.07144386 | 0.00362664 |
|  | Rothia_u_s | -0.0911225 | 0.00746286 | 0.00547613 |
| Cxcr1 | Aerococcus.urinaeequi | -0.1942031 | 0.01169405 | 0.00216288 |
|  | Aerococcus.viridans | -0.2758787 | 0.01661219 | 0.00216288 |
|  | Enterococcus.avium | 0.20919463 | 0.02847029 | 0.00216288 |
|  | Lactobacillus.sp..ASF360 | -0.526632 | 0.08148995 | 0.00422701 |
|  | Parabacteroides.goldsteinii | 0.45831685 | 0.10577072 | 0.04502628 |
|  | Rothia_u_s | -0.094801 | 0.0057085 | 0.00216288 |
| Cxcr2 | Lactobacillus.vaginalis | 0.12777597 | 0.01054324 | 0.00022944 |
|  | Parabacteroides.sp..20_3 | -0.09979 | 0.01170144 | 0.02746457 |
|  | Ruminococcus.bromii | -0.143145 | 0.01678527 | 0.02746457 |
|  | Turicibacter_u_s | -0.5374131 | 0.06301741 | 0.02746457 |
| Cxcr5 | Aerococcus.urinaeequi | -0.1905239 | 0.0077582 | 0.00058883 |
|  | Aerococcus.viridans | -0.2706521 | 0.01102104 | 0.00058883 |
|  | Enterococcus.avium | 0.20225404 | 0.03437628 | 0.00822736 |
|  | Lactobacillus.sp..ASF360 | -0.5357996 | 0.07364189 | 0.00234025 |
|  | Parabacteroides.goldsteinii | 0.46680291 | 0.1013255 | 0.03191549 |
|  | Rothia_u_s | -0.093005 | 0.00378719 | 0.00058883 |
| Faslg | Enterococcus.avium | 0.21983996 | 0.01548081 | 6.53E-05 |
|  | Firmicutes.bacterium.ASF500 | -0.4891914 | 0.07119854 | 0.03686321 |
|  | Lactobacillus.sp..ASF360 | -0.512857 | 0.09181764 | 0.02879847 |
| Il10ra | Aerococcus.urinaeequi | -0.190839 | 0.00779992 | 0.00059754 |
|  | Aerococcus.viridans | -0.2710998 | 0.01108031 | 0.00059754 |
|  | Enterococcus.avium | 0.20246786 | 0.03420277 | 0.00785984 |
|  | Lactobacillus.sp..ASF360 | -0.5337324 | 0.07549499 | 0.00286366 |
|  | Parabacteroides.goldsteinii | 0.48022626 | 0.09303211 | 0.01577087 |
|  | Rothia_u_s | -0.0931588 | 0.00380756 | 0.00059754 |
| IL11 | Aerococcus.urinaeequi | -0.1949758 | 0.00019233 | 2.08E-10 |
|  | Aerococcus.viridans | -0.2769762 | 0.00027322 | 2.08E-10 |
|  | Enterococcus.avium | 0.2031511 | 0.03349717 | 0.00662128 |
|  | Lactobacillus.sp..ASF360 | -0.535676 | 0.07375418 | 0.00239112 |
|  | Parabacteroides.goldsteinii | 0.46190777 | 0.10379976 | 0.03922108 |
|  | Rothia_u_s | -0.0951782 | 9.39E-05 | 2.08E-10 |
| Il13 | Aerococcus.urinaeequi | -0.1946808 | 0.00088077 | 9.21E-08 |
|  | Aerococcus.viridans | -0.2765573 | 0.00125119 | 9.21E-08 |
|  | Enterococcus.avium | 0.20325407 | 0.03341899 | 0.00649612 |
|  | Lactobacillus.sp..ASF360 | -0.5367141 | 0.07280468 | 0.0021524 |
|  | Parabacteroides.goldsteinii | 0.46289245 | 0.10324999 | 0.03752915 |
|  | Rothia_u_s | -0.0950342 | 0.00042995 | 9.21E-08 |
| Il16 | Aerococcus.urinaeequi | -0.1944225 | 0.00167265 | 1.19E-06 |
|  | Aerococcus.viridans | -0.2761904 | 0.00237612 | 1.19E-06 |
|  | Enterococcus.avium | 0.20254487 | 0.03395296 | 0.00732721 |
|  | Lactobacillus.sp..ASF360 | -0.5353304 | 0.07406717 | 0.00245226 |
|  | Parabacteroides.goldsteinii | 0.46467983 | 0.10224148 | 0.03427918 |
|  | Rothia_u_s | -0.0949081 | 0.00081651 | 1.19E-06 |
| Il17a | Aerococcus.urinaeequi | -0.2120692 | 0.01492029 | 0.04116187 |
|  | Aerococcus.viridans | -0.3012586 | 0.02119529 | 0.04116187 |
|  | Alistipes.indistinctus | 0.1901186 | 0.00775184 | 3.32E-05 |
|  | Bacteroides.fragilis | 0.10493896 | 0.007281 | 0.04116187 |
|  | Bifidobacterium.bifidum | -0.0386334 | 0.00269519 | 0.04116187 |
|  | Enterococcus.avium | 0.22037164 | 0.04587969 | 0.04116187 |
|  | Intestinimonas.butyriciproducens | -0.1493202 | 0.01050554 | 0.04116187 |
|  | Lactobacillus.sp..ASF360 | -0.6176657 | 0.04701655 | 0.02303498 |
|  | Parabacteroides.goldsteinii | 0.53529751 | 0.11274966 | 0.04116187 |
|  | Parabacteroides.sp..D13 | 0.34918276 | 0.07185384 | 0.04116187 |
|  | Porphyromonas.sp..31_2 | 0.3463533 | 0.07781523 | 0.04116187 |
|  | Rothia_u_s | -0.1035224 | 0.0072834 | 0.04116187 |
|  | Ruminococcaceae.bacterium.D16 | -0.1821023 | 0.0127156 | 0.04116187 |
| Il17b | Lactobacillus.vaginalis | -0.1560622 | 0.00730645 | 0.0004546 |
| Il1b | Lactobacillus.vaginalis | 0.12121433 | 0.01782808 | 0.01517494 |
| Il27 | Enterorhabdus_u_s | 0.19373258 | 0.02772109 | 0.01810406 |
| Il2rb | Lactobacillus.sp..ASF360 | -0.5179584 | 0.08816552 | 0.02028299 |
|  | Parabacteroides.goldsteinii | 0.51806 | 0.06241769 | 0.00364866 |
| Il3 | Aerococcus.urinaeequi | -0.2180188 | 4.98E-05 | 8.19E-07 |
|  | Aerococcus.viridans | -0.3097104 | 7.07E-05 | 8.19E-07 |
|  | Enterococcus.avium | 0.23902817 | 3.25E-05 | 2.34E-20 |
|  | Firmicutes.bacterium.ASF500 | -0.5333483 | 0.09197182 | 0.01499204 |
|  | Firmicutes.bacterium.M10.2 | -0.2226338 | 5.08E-05 | 8.19E-07 |
|  | Lactobacillus.animalis | -0.1064268 | 2.43E-05 | 8.19E-07 |
|  | Lactobacillus.sp..ASF360 | -0.6033732 | 0.09200126 | 0.00827436 |
|  | Parabacteroides.goldsteinii | 0.55259618 | 7.51E-05 | 2.34E-20 |
|  | Rothia_u_s | -0.1064267 | 2.43E-05 | 8.19E-07 |
| Il33 | Clostridiales_u_s | -0.5503931 | 0.06423834 | 0.04419003 |
|  | Lactobacillus.vaginalis | 0.11546167 | 0.0220914 | 0.04419003 |
| Il4 | Aerococcus.urinaeequi | -0.194643 | 0.00129196 | 4.19E-07 |
|  | Aerococcus.viridans | -0.2765035 | 0.00183532 | 4.19E-07 |
|  | Enterococcus.avium | 0.20427598 | 0.03263084 | 0.00524896 |
|  | Lactobacillus.sp..ASF360 | -0.5374943 | 0.07208158 | 0.00194886 |
|  | Parabacteroides.goldsteinii | 0.46050507 | 0.10457592 | 0.04096689 |
|  | Rothia_u_s | -0.0950157 | 0.00063068 | 4.19E-07 |
| Il6st | Parabacteroides.goldsteinii | 0.52789004 | 0.05109552 | 0.00072493 |
| Il7 | Enterococcus.avium | 0.22349718 | 0.00596585 | 5.84E-08 |
|  | Firmicutes.bacterium.ASF500 | -0.503968 | 0.08147512 | 0.03222468 |
|  | Lactobacillus.sp..ASF360 | -0.5164316 | 0.0892781 | 0.02226848 |
| Lfng | Bacteroides.caccae | 0.59280746 | 0.03808217 | 0.00804434 |
|  | Bacteroides.vulgatus | 0.57023026 | 0.1205711 | 0.04044044 |
|  | Enterococcus.avium | 0.20474603 | 0.03226049 | 0.00804434 |
|  | Lactobacillus.sp..ASF360 | -0.5254005 | 0.08247767 | 0.00804434 |
| Pf4 | Lactobacillus.vaginalis | 0.11972494 | 0.01874123 | 0.02552998 |
| RGD1561905 | Aerococcus.urinaeequi | -0.20264 | 0.00886973 | 0.00334601 |
|  | Aerococcus.viridans | -0.2878638 | 0.01260005 | 0.00334601 |
|  | Bacteroides.fragilis | 0.09940395 | 0.00432727 | 0.00334601 |
|  | Bifidobacterium.bifidum | -0.0367123 | 0.00160162 | 0.00334601 |
|  | Enterococcus.avium | 0.21451665 | 0.03622213 | 0.00921145 |
|  | Lactobacillus.sp..ASF360 | -0.5929832 | 0.03690448 | 0.00296103 |
|  | Parabacteroides.goldsteinii | 0.50017128 | 0.10565841 | 0.02335309 |
|  | Parabacteroides.sp..D13 | 0.3305538 | 0.05779499 | 0.01852166 |
|  | Porphyromonas.sp..31_2 | 0.33800622 | 0.06194217 | 0.01305005 |
|  | Rothia_u_s | -0.0989195 | 0.00432979 | 0.00334601 |
| Tnfrsf11b | Bacteroides.intestinalis | 0.48182393 | 0.08936694 | 0.01794616 |
|  | Bifidobacterium.bifidum | -0.259337 | 0.01757437 | 0.00438312 |
|  | Enterococcus.faecium | -0.2926325 | 1.15E-05 | 7.93E-16 |
|  | Imtechella.halotolerans | -0.3915736 | 1.54E-05 | 7.93E-16 |
| Tnfsf14 | Lachnospiraceae.bacterium.3.1 | -0.5964908 | 0.09665719 | 0.02944834 |
| Tnfsf4 | Parabacteroides.goldsteinii | 0.51998756 | 0.05909246 | 0.00362087 |
| Vegfa | Enterococcus.avium | 0.22139977 | 0.01239453 | 1.07E-05 |
